# “Armed *in-vitro* retina”-generating microglial retinal organoids, where are we now?

**DOI:** 10.3389/fcell.2025.1574283

**Published:** 2025-05-30

**Authors:** Yaohong Liu, Lixiong Gao, Wenqian Chen, Yuhan Yan, Zi Ye, Zhaohui Li

**Affiliations:** ^1^ Department of Ophthalmology, Third Medical Center of Chinese PLA General Hospital, Beijing, China; ^2^ School of Medicine, Nankai University, Tianjin, China; ^3^ Chinese People's Liberation Army Medical School, Beijing, China

**Keywords:** retinal organoids, immune privilege, co-culture with immune cells, microglia, personalized treatment

## Abstract

The objective of organoid research is to develop *in vitro* models that accurately replicate the microenvironment of tissues and organs *in vivo*. Although techniques for culturing retinal organoids (ROs) have advanced significantly, they still fall short of incorporating all cell types necessary for maintaining retinal homeostasis, particularly immune cells like microglia. Standardizing the inclusion of immune cells in RO cultures would greatly enhance research into the mechanisms underlying retinal diseases and the discovery of therapeutic targets. This review examines recent advancements in co-culturing ROs with immune cells to mimic the physiological and pathological microenvironments of the retina, focusing on tissue structure and function. Furthermore, it emphasizes the importance of cutting-edge organoid technologies, such as microfluidics and organ-on-chip systems, in propelling research in this field. The goal is to equip researchers with a more profound understanding of microglial ROs and their potential applications in scientific investigations.

## 1 Introduction

### 1.1 Generation of organoids

Current disease research heavily relies on animal models and human-derived cells. However, the limited availability of human-derived cells means that our understanding of retinal pathophysiology primarily stems from studies using animal models (Watson and Lako, 2023). There are significant differences between animal and human cells, which prevent animal models from fully replicating the onset and progression of human diseases. For example, retinal resident immune cells, such as microglia, exhibit distinct mechanisms in regulating neuroinflammation in humans compared to mice (Edler et al., 2021). When induced by IFNγ, human microglia upregulate the expression of human leukocyte antigen, a process that is not inhibited by TGFβ1 (Smith et al., 2013). In contrast, mouse microglia increase the expression of major histocompatibility complex II (MHC II) proteins, a process that is suppressed by TGFβ1 (O'Keefe et al., 1999). This underscores the advantages of tissue models that mimic the human retinal microenvironment over animal models, which inherently possess physiological and functional differences. Such considerations have spurred the development of organoids as a superior research tool.

Organoids can be derived from either adult stem cells (aSCs) or pluripotent stem cells (PSCs) (Hautefort et al., 2022; Cameron et al., 2024), each with distinct preparation methods and characteristics. Organoids derived from aSCs are generated from tissue samples obtained from healthy individuals or patients, preserving the genetic and epigenetic backgrounds of the donors to a significant extent (Treveil et al., 2020). Conversely, PSC-derived organoids are developed from embryonic pluripotent stem cells (ePSCs) or induced pluripotent stem cells (iPSCs), and they tend to exhibit less specific retention of donor characteristics (Jowett et al., 2022). Unlike *ex vivo* organ cultures obtained through biopsies or resections, which have limited lifespans due to the absence of blood supply, organoids grown using 2D or 3D culture techniques can self-renew, self-organize, and differentiate into various cell types (Choi et al., 2023). These organoids form miniature organ-like structures that partially recapitulate the cellular composition, spatial organization, and functions of real organs, making them an ideal model for studying pathophysiological changes in human tissues and organs (Papp et al., 2024). Additionally, the development of such *in vitro* alternatives significantly reduces the number of animals used in preclinical research and offers potential applications in drug screening, organ transplantation, and personalized therapies. Existing studies have successfully developed organoid models for various organs, including the brain (Smirnova and Hartung, 2024; Andrews and Kriegstein, 2022), heart (Sahara, 2023; Richards et al., 2020), lungs (Miller et al., 2019; Joo et al., 2024), liver (Fang et al., 2024; Liu et al., 2023), kidneys (Yousef Yengej et al., 2020; Grassi et al., 2019), pancreas (Casamitjana et al., 2022; Chen et al., 2024), stomach (Chakrabarti et al., 2021; Cherne et al., 2021), and intestines (Read et al., 2022; Serra et al., 2019). This article focuses specifically on retinal organoids (ROs) for detailed discussion.

In 2011, Japanese scientist Eiraku successfully induced ePSCs to generate 3D-ROs for the first time (Eiraku et al., 2011). Following this groundbreaking study, the culture conditions and differentiation efficiency of ROs have been continuously refined. These advancements have led to the development of ROs containing photoreceptor cells, thereby enhancing their functional complexity (Pan et al., 2020). Furthermore, tissue structures that better simulate the retinal microenvironment have been increasingly integrated into these models in recent years (Zhang and Jin, 2021; Cowan et al., 2020; Kim et al., 2019). The creation of organoids represents a revolutionary platform for studying human retinal diseases and development. ROs not only replicate the retinal developmental process but also exhibit specific pathological features of diseases, providing an experimental model that closely mimics the real microenvironment *in vivo* (Usui-Ouchi et al., 2023).

### 1.2 Characteristics of ROs

The foundation of ROs typically originates from PSCs. By applying specific signaling molecules to PSC “seeds”, these cells can be induced to differentiate into neuroectodermal tissues and self-organize into organoids. This process is driven by the adhesive forces of retinal progenitor cells (RPCs) and actomyosin-mediated mechanical forces, ultimately resulting in the formation of well-structured organoids (Lowe et al., 2016). The differentiation of RPCs within ROs is further regulated by specific genes and signaling pathways. For instance, research by Cuevas et al. demonstrated that editing the NRL gene can guide RPCs to differentiate into S-cone-like cells while inhibiting their differentiation into rod cells, thereby highlighting the critical role of genetic switches in organoid differentiation (Cuevas et al., 2021). Additionally, Brooks et al. showed that the inclusion of factors such as docosahexaenoic acid and fibroblast growth factor 1 significantly enhances the maturation of photoreceptors, including cone cells (Brooks et al., 2019). Current RO culture methods can now effectively induce and enrich specific retinal cell types (Bell et al., 2020; Chew et al., 2022), such as photoreceptors, retinal ganglion cells (RGCs), bipolar cells, horizontal cells, astrocytes, and Müller glia (Zhang and Jin, 2021; Sun et al., 2023; Dorgau et al., 2022).

Photoreceptors, including rods and cones, are essential for capturing photons and converting them into electrical signals. Rod cells facilitate black-and-white vision in low-light conditions, while cone cells enable color vision in bright light (Nazarenko and Didenko, 2023). Successfully inducing photoreceptors is vital for studying retinal phototransduction mechanisms and developing treatments for degenerative retinal diseases (Hussey et al., 2022). RGCs serve as the output neurons of the retina, with their axons forming the optic nerve to transmit visual information to the brain’s visual centers (Sanie-Jahromi et al., 2022). The successful differentiation of RGCs is crucial for researching optic nerve diseases, such as glaucoma, and can also provide a model for neural regeneration studies (Subramani et al., 2023). Bipolar cells, the intermediate neurons of the retina, connect photoreceptors and RGCs, integrating signals from photoreceptors and relaying them to RGCs (Ganzen et al., 2024). The presence of bipolar cells in ROs is essential for understanding visual signal pathways and the transmission of photoelectric signals (Ichinose and Habib, 2022). Horizontal cells establish lateral connections among photoreceptors, regulating retinal light adaptation and contrast sensitivity. Their inclusion enhances the comprehensiveness of functional studies on retinal neural networks (Castillo García and Urdapilleta, 2022). Astrocytes and Müller cells, both types of retinal glial cells, play a role in regulating neuronal metabolism within the retina, contributing to a more systematic understanding of the mechanisms underlying retinal diseases (Shinozaki et al., 2023; Chen et al., 2022).

To enrich the diversity of cell types and better simulate the retinal environment, some research teams have developed co-culture systems that combine ROs with retinal pigment epithelium (RPE) cells (Akhtar et al., 2019; Su et al., 2022; Mathivanan et al., 2015). Notably, the inclusion of RPE cells has been shown to promote the enrichment and accelerate the maturation of photoreceptor progenitor cells, underscoring the importance of cellular and structural diversity in establishing successful *in vitro* models. In addition to exogenous co-culture methods, ROs containing RPE cells can also be generated through spontaneous differentiation. In 2012, Nakano’s team published a study demonstrating that ePSCs, when cultured in a three-dimensional system, could spontaneously form optic vesicle-like structures and further differentiate into functional RPE cells (Nakano et al., 2012).

Currently, existing RO models cannot fully replicate the complexity of the *in vivo* environment (Fathi et al., 2021). The potential for incorporating other missing cell types and structures through similar co-culture techniques, as well as the possibility of achieving functional restoration, remains an area for further exploration (Martinelli et al., 2022).

### 1.3 Limitations of current RO culture

Due to the directed differentiation process, ROs are derived from neuroectoderm and lack various cell types that originate from different germ layers, such as mesoderm-derived vascular endothelial cells and yolk sac-derived microglia (Ginhoux et al., 2010; Kierdorf et al., 2013; Schulz et al., 2012; Hoeffel and Ginhoux, 2018). Vascular endothelial cells are essential components of retinal blood vessels, responsible for delivering oxygen and nutrients to photoreceptors and other retinal neurons. The absence of a vascular system in organoids can restrict the diffusion of oxygen and nutrients, leading to reduced cellular function or even cell death in deeper layers (Zhao et al., 2021a). Moreover, vascular endothelial cells are crucial for forming the blood-retinal barrier (BRB). Without these cells, ROs cannot replicate the functional properties of the BRB, which significantly limits the study of related diseases (O'Leary and Campbell, 2023).

In addition to vascular endothelial cells, microglia play an indispensable role in maintaining retinal homeostasis. As the predominant immune cells in the retina, microglia monitor neural tissue health, clear debris, and regulate inflammatory responses under normal physiological conditions. The lack of microglia in ROs complicates the replication of the immune environment and responses observed *in vivo*. This deficiency not only impedes the differentiation and maturation of other retinal cells (Taylor et al., 2020; Noel et al., 2017) but may also contribute to the limited long-term viability of inner RO layers (Sridhar et al., 2020). By incorporating immune cells, organoids can more accurately mimic the immune responses of the retina under both physiological and pathological conditions, including neuroprotection, inflammation, and tissue repair processes. Unlike traditional ROs, microglial organoids can be utilized to model immune rejection in host-graft interactions, screen immunomodulatory drugs, and assess the safety of immunotherapies.

The retina is a unique immune-privileged site, characterized by a specialized immune state that entails distinct immune response mechanisms. The co-culture of immune cells, particularly microglia, with traditional ROs, along with the methodological and functional validation of this approach, has emerged as a significant focus of current research.

## 2 Immune status of the retina

### 2.1 Mechanisms of “immune privilege” in the retina

The eye exhibits a distinctive immune state referred to as “immune privilege”, which enables it to evade robust immune responses when exposed to antigens. This mechanism is crucial for maintaining the transparency of the visual axis and safeguarding vision (Nieto-Aristizábal et al., 2022). It is underpinned by both structural and functional elements.

Structurally, the blood-ocular barrier acts as a protective barrier, formed by the non-fenestrated endothelial cells of the iris and ciliary body, along with the retinal vascular endothelium and RPE. Together, the latter two components create the BRB, which effectively restricts the infiltration of immune cells and inflammatory agents. Furthermore, the absence of a direct lymphatic system in the eye inhibits the systemic recognition of antigens.

Functionally, the eye synthesizes immunomodulatory factors such as IL-10, TGF-β, and PD-L1, which suppress the activation of antigen-presenting cells (APCs) and effector T cells, thereby reducing inflammatory responses. Given the retina’s highly sensitive neural tissue and complex photoreceptor network, it necessitates special protection to avert immune-mediated damage, making it a vital aspect of the eye’s immune privilege (Keino et al., 2018).

While these mechanisms shield the eye from inflammation due to external pathogens, autoimmune reactions, and trauma, they are also essential in corneal and RPE transplantation, as well as in managing autoimmune uveitis (Niederkorn, 2019). However, the immunosuppressive nature of this “barrier” can become a double-edged sword in chronic infections, such as viral retinitis, and in cases of tumor growth or invasion. In such scenarios, pathogens and tumor cells may escape swift recognition and elimination, adversely affecting disease prognosis. Additionally, under pathological conditions, the immune privilege of the retina may be compromised, leading to the infiltration of inflammatory cells and factors, which can result in retinal tissue damage and neurodegenerative changes (Qiao et al., 2009).

### 2.2 Resident immune cells in the physiological state

The retina contains a variety of resident immune cells, with microglia being the most prominent. Additionally, smaller populations of dendritic cells, astrocytes, Müller cells, and macrophages are present (Fan et al., 2022). As an extension of the central nervous system (CNS), microglia function as resident immune cells in both the CNS and the retina (Usui-Ouchi et al., 2023). In the adult eye, microglial renewal primarily occurs through self-proliferation; however, under certain conditions, bone marrow-derived cells can cross the blood-brain barrier (BBB) or blood-retinal barrier (BRB) into the CNS and differentiate into microglia (Jin et al., 2017). In a healthy eye, microglia constitute approximately 0.3%–1.0% of retinal cells, performing essential functions such as immune surveillance, synaptic remodeling, neurotrophic support, vascular development, and debris clearance (Lukowski et al., 2019; Silverman and Wong, 2018). Despite their relatively low abundance, microglia are increasingly recognized as crucial players in the pathogenesis of ocular diseases.

Under steady-state conditions, retinal microglia are mainly localized in the inner plexiform layer (IPL) and outer plexiform layer (OPL) (Santos et al., 2008), exhibiting a highly branched morphology and long-term motility that enables them to dynamically monitor the ocular environment (Silverman and Wong, 2018). In response to localized injury, infection, or hypoxia, microglia become activated and migrate purposefully to the outer nuclear layer (ONL), RPE, and subretinal space (Usui-Ouchi et al., 2020). This activation is associated with a decline in neurotrophic functions, increased cytokine secretion, and heightened phagocytic activity (Cherry et al., 2014; Brown and Neher, 2014). The cytokines released can induce resident glial cells to release neurotoxins and recruit immune cells from outside the eye. Furthermore, cytokines mediate crosstalk between microglia and Müller glia, playing a critical role in regulating the adaptive response to retinal injury (Wang et al., 2011).

Dendritic cells primarily originate from bone marrow progenitors and migrate to the retina via blood circulation during embryonic development, where they differentiate into dendritic cells in response to local environmental stimuli (Ohteki et al., 2021). These cells are predominantly located in the nerve fiber layer (Zhao and Yu, 2024) and are involved in antigen presentation and initiating immune responses by expressing MHC II. Under physiological conditions, their expression remains low to prevent unnecessary inflammation and maintain the immune privilege of the retina (Xu et al., 2007). Astrocytes, which originate from neuroepithelial progenitor cells, migrate into the retina along optic nerve axons during embryonic development, gradually populating the nerve fiber layer and providing structural support for the vascular network and neurons (Zheng et al., 2022). Müller cells, derived from RPCs within the optic cup, are among the last cell types to differentiate in the retina, functioning as a “neuronal scaffold” (Gao et al., 2021). Both astrocytes and Müller cells help regulate immune and inflammatory responses by secreting TGF-β and other anti-inflammatory molecules to suppress excessive activation of T cells and microglia, thereby protecting neurons from damage (Mochizuki et al., 2013). They also play a crucial role in maintaining the integrity of the BRB, limiting the entry of external pathogens and inflammatory factors into retinal tissue (He et al., 2024). Meanwhile, macrophages residing in the outer retinal and choroidal layers act as “cleaners”, responsible for clearing cellular debris and maintaining retinal homeostasis (McMenamin et al., 2019). During embryonic development, macrophages originate from primitive hematopoietic stem cells (HSCs) in the yolk sac and migrate to the retina as primitive macrophages. After colonization, they further differentiate into microglia and resident macrophages (Wu and Hirschi, 2020). Postnatally, retinal macrophages can also arise from monocytes derived from the bone marrow (Fige et al., 2022).

### 2.3 External immune cells under pathological conditions

Mechanical injuries, infections, immune-mediated inflammation, ischemia, and tumors can disrupt the BRB and compromise immune privilege, facilitating the infiltration of external immune cells, including neutrophils, monocytes, T cells and B cells, into the retina (He et al., 2024). This recruitment and the process of crossing the barrier are regulated by various factors. For instance, during retinal inflammation, macrophages located near retinal blood vessels release chemokines that attract external immune cells (Sterling et al., 2024). Furthermore, the upregulation of adhesion molecules such as ICAM-1 and P-selectin on vascular endothelial cells enhances the adhesion and migration of immune cells (Xu et al., 2003). Pro-inflammatory cytokines like TNF-α and IL-1β increase BRB permeability by modulating the expression of tight junction proteins in vascular endothelial cells (Bamforth et al., 1997). In certain instances, RPE may serve as a “gateway” for immune cell entry, as studies indicate that RPE expresses adhesion molecules like VCAM-1, allowing monocytes to adhere and migrate into retinal tissue (Benhar et al., 2016).

The types of immune cells recruited differ across various pathological conditions. Mechanical injuries, such as ocular trauma or retinal reattachment surgery, predominantly attract neutrophils and macrophages (Azzam et al., 2024). Inflammatory responses, including infectious uveitis caused by bacteria, fungi, viruses, or parasites (Zinkernagel et al., 2013), as well as autoimmune uveitis (Okunuki et al., 2019), can lead to the infiltration of T cells, B cells, macrophages, and neutrophils into the retina. These infiltrating immune cells exacerbate inflammation and tissue damage by releasing pro-inflammatory cytokines such as IFN-γ and IL-1β. In systemic autoimmune diseases like systemic lupus erythematosus or Sjögren’s syndrome, immune hyperactivation can induce vasculitis and immune complex deposition, disrupting the retinal vascular network and causing vascular leakage and immune cell infiltration (McMenamin et al., 2019). In diabetic retinopathy (DR), hyperglycemia triggers a low-grade chronic inflammatory response that gradually damages the BRB, facilitating the slow infiltration of monocytes and neutrophils into the retina. This infiltration, coupled with the release of pro-inflammatory factors like TNF-α and IL-6, exacerbates microvascular injury and promotes neovascularization (Wang et al., 2023). These newly formed blood vessels are often accompanied by further immune cell infiltration (Karlstetter et al., 2015). Tumors can also compromise the BRB, eliciting external immune responses (Zhu et al., 2023). For instance, in retinoblastoma (RB), external immune cells such as T cells, natural killer (NK) cells, and macrophages infiltrate the retina to target tumor cells, thereby limiting tumor growth and spread (Pascual-Pasto et al., 2024).

**FIGURE 1:**
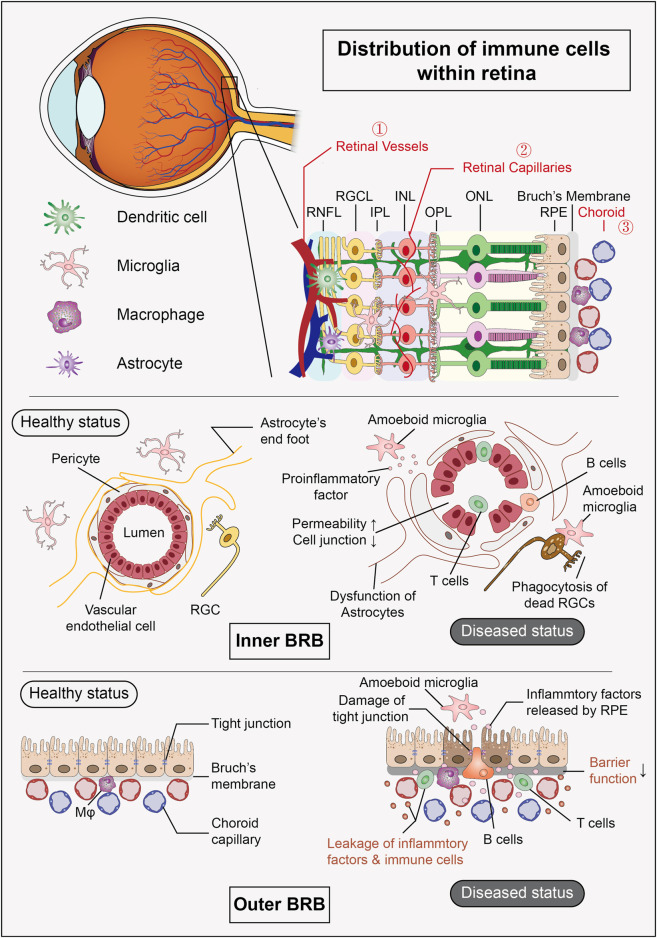
Distribution of immune cells within retina and composition of BRB. The retina harbors a group of resident immune cells that work together to maintain the stability of the internal environment. Additionally, the retinal vessels and their surrounding tissues form the inner BRB, while the RPE and choroid create the outer BRB, both of which provide structural support for the eye’s “immune privilege” under physiological conditions. (RNFL: retinal nerve fiber layer, RGCL: retinal ganglion cell layer, IPL: inner plexiform layer, INL: inner nuclear layer, OPL: outer plexiform layer, ONL: outer nuclear layer, RPE: retinal pigment epithelium, BRB: blood-retinal barrier).

In retinal diseases, infiltrating immune cells and resident immune cells collaborate, collectively driving the onset and progression of the disease (Figure 1).

## 3 Related retinal diseases involving immune cells

### 3.1 Related retinal diseases involving microglia

In the retina, microglia serve as the primary resident immune cells (Fan et al., 2022), continuously monitoring their environment and interacting with other retinal cells to uphold microenvironmental homeostasis. Under normal physiological conditions, they offer immune protection to the posterior eye, including the neural retina. When stimulated externally, microglia are initially activated to induce inflammation. As the process of retinal repair advances, they shift to a reparative, anti-inflammatory state, skillfully balancing pro-inflammatory and anti-inflammatory responses to promote tissue healing (Patel and Lamba, 2023). However, in pathological conditions, microglia may become depleted or excessively activated, leading to the production of pro-inflammatory neurotoxic cytokines or pro-angiogenic factors. They may also phagocytose viable neural cells, resulting in neurofunctional impairment (Ryan et al., 2023). Microglia have been recognized as key players in the onset and progression of various retinal diseases (Silverman and Wong, 2018; Rathnasamy et al., 2019), such as age-related macular degeneration (AMD) (Rashid et al., 2019; Fletcher, 2020), retinitis pigmentosa (RP) (O'Koren et al., 2019; Gallenga et al., 2021), uveitis (Rashid et al., 2019; Huang et al., 2024), DR (Jin et al., 2017; Marcinkowska et al., 2022), retinal vein occlusion (RVO) (Marcinkowska et al., 2022; Tang et al., 2022), and RB (Xu et al., 2022; Barresi et al., 2024).

#### 3.1.1 Degeneration-related diseases

AMD is a chronic degenerative retinal disease predominantly found in individuals over 50 years old. It ranks as a leading cause of vision loss among older adults and is marked by progressive macular atrophy or choroidal neovascularization (CNV) in the macular region, observable through fundoscopic examination. Pathological findings indicate that degeneration primarily affects the RPE, Bruch’s membrane, and the photoreceptor layer (Barresi et al., 2024). Early research has demonstrated that microglia accumulate in the subretinal space of AMD patients, particularly in regions of retinal degeneration and CNV. These microglia exhibit rhodopsin-positive cytoplasmic inclusions, suggesting they have phagocytosed debris from rod photoreceptors (Zhang and Wong, 2021). However, instead of protecting the retina, this process may worsen tissue damage by harming neighboring photoreceptors. The debris can activate microglia directly, further escalating retinal inflammation and advancing geographic atrophy in the macula (Gupta et al., 2003). Beyond phagocytosis, microglia also contribute to photoreceptor death by releasing pro-inflammatory cytokines such as TNF-α and IL-1β, along with complement component C3 (Zabel et al., 2016; Bravo-Gil et al., 2017). Collectively, these findings underscore that microglial dysfunction may play a pivotal role in exacerbating retinal degeneration and inflammation.

RP is a hereditary retinal degenerative disease, most often inherited in an autosomal recessive manner (Bravo-Gil et al., 2017), with a global prevalence of approximately one in 4,000 individuals (Verbakel et al., 2018). Clinically, RP is characterized by night blindness, progressive narrowing of the visual field, and eventual loss of central vision. Its primary pathological features include the degeneration of photoreceptor cells and RPE, along with the distinctive “bone spicule” pigmentation (Beryozkin et al., 2020). In RP, microglia express various chemokine receptors, facilitating the mobilization and recruitment of monocytes through chemokine-receptor interactions (Rutar et al., 2015). In Sennlaub’s research, the CX3CR1/CX3CL1 signaling pathway is crucial for neuron protection and immune homeostasis in the retina under normal conditions. In aged CX3CR1-deficient mice, however, microglia abnormally accumulate in the subretinal space, leading to photoreceptor degeneration (Sennlaub et al., 2013). Studies have also indicated that in mouse models of Stargardt disease and RP, retinal microglia-produced CCL3 (MIP-1α) can worsen inflammation and degeneration (Kohno et al., 2014). Research by Zhao and colleagues revealed that in the rd10 model of RP, microglia accelerate retinal degeneration by phagocytosing rod photoreceptors. This suggests that inhibiting the phagocytic activity of microglia could slow disease progression and offer neuroprotective benefits in certain degenerative retinal diseases (Zhao et al., 2015). By transplanting C-Kit+/SSEA4-RPCs into models of retinal degeneration, Zou’s team discovered that microglial activation can be significantly inhibited. This inhibition reduces gliosis and the production of inflammatory mediators, fostering a healthier microenvironment for transplanted cells and slowing the progression of retinal degenerative diseases (Zou et al., 2019).

**FIGURE 2:**
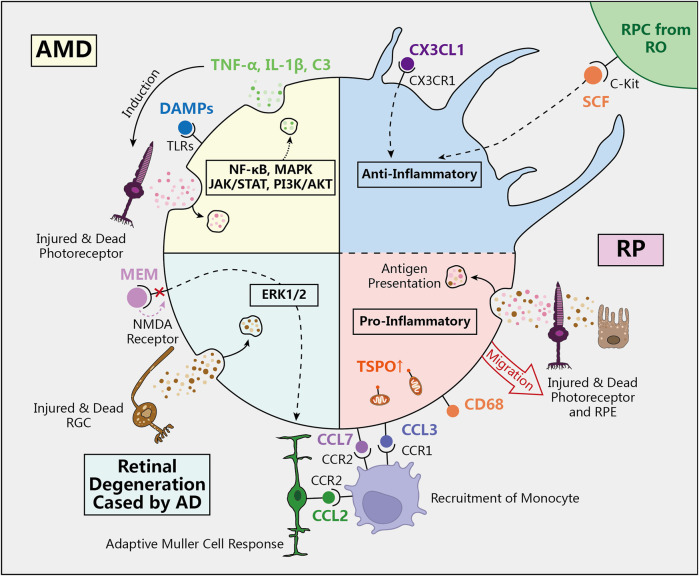
The role of microglia in retinal degeneration diseases. In physiological conditions, quiescent microglia in ramified shape regulate the immune environment to prevent excessive inflammatory damage to the retina. However, in AMD, RP, and retinal degeneration caused by AD, activated microglia transform into amoeboid shape and participate in the inflammatory response through various signaling pathways and molecular mechanisms. (AMD: age-related macular degeneration, RP: retinitis pigmentosa, AD: Alzheimer’s Disease, SCF: stem cell facter, DAMPs: Damage-associated molecular patterns, TLRs: Toll-like receptors, MEM: memantine, NMDA: Nmethyl-D-aspartate, TSPO: translocator protein).

Alzheimer’s Disease (AD) is a neurodegenerative disorder characterized by progressive cognitive decline. Beyond its effects on the central nervous system, it can also lead to retinal degeneration, resulting in a decrease in RGC numbers. Clinically, this is evident through symptoms such as vision loss and visual field defects. In some instances, retinal abnormalities may manifest before the clinical symptoms of AD, positioning them as potential early biomarkers for AD screening (Araya-Arriagada et al., 2021; Liao et al., 2021). In our previous studies, we observed retinal degeneration in the AD animal model APPswe/PS1ΔE9 double-transgenic mice, primarily characterized by RGC loss and microglial activation. The Nmethyl-D-aspartate (NMDA) receptor antagonist memantine (MEM), a treatment for AD, has been shown to exert neuroprotective effects on RGCs by inhibiting activated microglia in the retina and modulating Müller cell responses (Gao et al., 2015). (Figure 2)

Currently, most mechanistic studies on microglia are conducted using animal models. However, certain genetic risk variants associated with retinal degeneration, such as HTRA1, C2, and C3, are exclusively expressed in human microglia (Gosselin et al., 2017). Therefore, utilizing human-derived retinal organoids to simulate the retinal environment is crucial for understanding the unique characteristics and pathological responses of human microglia.

#### 3.1.2 Autoimmune-related diseases

Abnormal activation of the immune system can disrupt immune balance, leading to inflammation that targets the retina and choroid (Wang et al., 2023). This process is typically mediated by immune cells, with microglia from various origins potentially playing opposing roles. Previous research has shown that resident microglia tend to differentiate into the M1 phenotype, which exhibits pro-inflammatory and neurotoxic characteristics. In contrast, bone marrow-derived microglia are more likely to differentiate into the M2 phenotype, providing protective effects on retinal neurons (Jin et al., 2017; Jin et al., 2021).

Uveitis is a significant cause of blindness and can also indicate systemic diseases. Retinal microglia play a crucial role in the development of uveitis. Endotoxin-induced uveitis, a well-established animal model of acute inflammatory uveitis, demonstrates that following lipopolysaccharide (LPS) injection, microglia are rapidly activated, infiltrate the photoreceptor layer, and migrate to the retinal vasculature (Couturier et al., 2014). Furthermore, in experimental autoimmune uveitis, the disease appears to be primarily mediated by microglia, as leukocytes cannot penetrate the BRB to infiltrate the retina (Okunuki et al., 2019).

Functional analysis of activated microglia reveals that their cell membranes express various antigen markers, such as leukocyte and macrophage antigens CD45 and CD68, indicating their potential role in antigen presentation (Penfold et al., 1991; Lipski et al., 2017). Additionally, microglia produce pro-inflammatory cytokines, including TNF-α, IL-1β, IL-6, and CCL2, as well as the toxic mediator nitric oxide (Sierra et al., 2014). Collectively, these mediators contribute to the breakdown of the BRB, recruitment of peripheral leukocytes, and permanent retinal damage (Colonna and Butovsky, 2017; Kitaoka et al., 2006).

#### 3.1.3 Ischemia-related diseases

The most prevalent ischemic retinal diseases include DR and RVO(136, 137). DR is a significant complication of diabetes, with its incidence rising over time. It is marked by microvascular abnormalities, chronic inflammation, and retinal neurodegeneration, making it a leading cause of vision impairment and blindness (Zhan, 2023). RVO, the second most common retinal vascular disease following DR, is categorized into central and branch types. Clinically, RVO is characterized by venous occlusion, retinal hemorrhage, and macular edema. Pathological changes associated with RVO include blood flow obstruction, hypoxia, and neovascularization (Yin et al., 2022).

In both animal models and human patients with DR, activation and infiltration of microglia have been observed (Zeng et al., 2008). Hyperglycemia directly stimulates microglia, enhancing the expression of cytokines such as IL-1β, TNF-α, and vascular endothelial growth factor (VEGF) (Kinuthia et al., 2020). The accumulation of mediators like aldose reductase (Chang et al., 2019), reactive dicarbonyls (Schlotterer et al., 2019), and advanced glycation end-products (Schlotterer et al., 2019) further contributes to microglial activation and intensifies inflammation. Additionally, the proangiogenic factor angiotensin II can directly activate the angiotensin type 1 receptor in microglia, playing a significant role in retinal inflammation linked to DR (144).

In RVO, overactivated microglia not only secrete pro-inflammatory cytokines but also release VEGF, which leads to pathological neovascularization, exacerbating retinal edema and vision loss (Checchin et al., 2006). During retinal ischemia/reperfusion injury, activated microglia upregulate the expression of C1q, contributing to retinal damage. Conversely, the absence of C1q has been shown to suppress microglial activation and proliferation, thereby protecting RGCs and improving visual function (Silverman et al., 2016). Furthermore, microglia are believed to interact with peripheral macrophages to regulate retinal hypoxia and maintain the integrity of the BRB(147). Through the CX3CR1 signaling pathway, microglia can also interact with vascular endothelial cells to regulate retinal vessel diameter and local blood supply (Mills et al., 2021).

#### 3.1.4 Tumor

RB is a malignant intraocular tumor predominantly affecting children, resulting from genetic mutations in RPCs. It is characterized by rapid tumor cell proliferation, aggressive invasion, and significant impairment of visual function (Cruz-Gálvez et al., 2022). Microglia play a pivotal role in both the initiation and progression of RB by modulating the tumor microenvironment. They contribute to local inflammation in tumor regions through the secretion of pro-inflammatory cytokines and factors, which in turn promote tumor growth and angiogenesis (Aires et al., 2020). The activation of microglia is often stimulated by signals such as exosomes and chemokines released by tumor cells, creating a complex interplay between the two (Wang and Cepko, 2022). Conversely, microglia can also mitigate tumor spread to some degree by phagocytosing necrotic tumor cells and their debris (Zhao et al., 2021b).

Their involvement extends beyond RB, as microglia are also crucial in other retinal tumors, including retinal melanoma (Murenu et al., 2022) and retinal lymphoma (Guo et al., 2022). As essential components of the tumor microenvironment, microglia present potential therapeutic targets. Modulating their activity or signaling pathways could lead to promising strategies for treating retinal tumors.

The advent of 3D microfluidic vascularized tumor organoid models has provided valuable tools for investigating the transport of immune cells during cancer progression. These models create endothelial-lined vascular networks within organoids, enabling researchers to observe the movement of T cells through the vascular network and their interactions with tumor spheroids *in vitro*. This innovative system opens new avenues for exploring tumor-induced immune response mechanisms and for the preclinical evaluation of the efficacy of combined immunotherapy and chemotherapy approaches (Zhao et al., 2024).

### 3.2 The role of other immune cells in retinal diseases

Dendritic cells represent a crucial category of resident immune cells within the retina. They are instrumental in immune activation during inflammatory retinal diseases and facilitate T cell infiltration into the retina, which can worsen inflammation (Xu et al., 2007). Similarly, a limited number of macrophages inhabit the retina and choroid. In conditions such as AMD and DR, these macrophages exacerbate disease progression by releasing inflammatory mediators like IL-6 and TNF-α(85). In cases of retinal ischemia-reperfusion injury and other inflammatory retinal diseases, Müller cells also play a significant role in immune activation and the enhancement of inflammatory responses. Furthermore, they work in conjunction with other retinal glial cells to establish a complex immune network, which may ultimately result in irreversible retinal damage (Mochizuki et al., 2013).

In conclusion, beyond microglia, various immune cells in the retina—including dendritic cells, macrophages, and Müller cells—are pivotal in the onset and advancement of retinal diseases. These cells engage in a sophisticated immune regulatory network that not only protects the retina but can also aggravate disease when immune regulation fails. Gaining a deeper understanding of the functions of these immune cells and targeting their activities may pave the way for innovative therapeutic strategies in the treatment of retinal diseases.

## 4 Attempts to construct microglial ROs

There is an increasing acknowledgment of the necessity to create *in vitro* systems that are more physiologically relevant and incorporate an “immune component”. The specific mechanisms through which microglia influence various retinal diseases remain ambiguous. Consequently, it is crucial to develop co-culture models that integrate immune cells, especially microglia, with ROs. Presently, established human RO models do not contain resident microglia within the retinal layers. By enhancing cell diversity in ROs through the incorporation or generation of retinal microglia, we can achieve a more comprehensive and accurate representation of the native retina. This enhancement would also facilitate improved modeling of diseases where microglia are integral, thereby opening avenues for the discovery of new therapeutic strategies.

### 4.1 The establishment of microglial ROs

#### 4.1.1 Exogenous addition of immune cells

Xu et al. utilized human embryonic stem cells (hESCs) for differentiation. After approximately 49 days of culture, microglial precursor cells were harvested from the culture supernatant and further cultivated in low-adhesion plates until day 56, resulting in the generation of mature microglia. Concurrently, another portion of hESCs was induced to form 3D-ROs through a transition from adherent to suspension culture. During this transition, the differentiated mature microglia were introduced into the organoids for co-culture. Throughout the subsequent cultivation period, microglia were observed to migrate into and integrate within the organoids, successfully maintaining viability for at least 60 days (Xu et al., 2024b).

Usui-Ouchi et al. developed an innovative 3D-RO model containing microglia by co-culturing retinal organoids with macrophage precursor cells (MPCs) derived from human induced pluripotent stem cells (hiPSCs). Building on previous studies, the team further optimized the parameters for the successful integration of MPCs into the organoids. They found that the survival of MPCs depended on macrophage colony-stimulating factor, while the addition of other factors, such as Tgfb1 or CX3CL1, did not enhance MPC integration into the organoids. Under optimized co-culture conditions, CD45-positive and IBA1-positive cells were detected within the retinal layers and lumen of the organoids after just 2 weeks. The results indicated that in the presence of reactive oxygen species, MPCs migrated to the OPL, the corresponding location of microglia in healthy retinal tissue, and developed a mature morphology characterized by small cell bodies with long branching processes—features typically observed only *in vivo*. Once induced microglia (iMG) were stably localized in the OPL, they transitioned from an initial pro-inflammatory state at week two to a physiological state by week 6, as evidenced by the downregulation of pro-inflammatory cytokines and upregulation of anti-inflammatory cytokines. This progression underscores that after an initial activation phase, the iMG entered a stable and mature microglial stage (Usui-Ouchi et al., 2023).

Gao et al. derived iMG from human HSCs and noted that the retinal organoid microenvironment significantly enhanced the functionality and organelle maturation of iMG during co-culture. Notably, compared to HMC3, a commonly used immortalized human microglial cell line that retains most primary microglial characteristics, iMG expressed higher levels of typical microglial markers and exhibited an immune response profile closely resembling that of primary human microglia. This finding establishes iMG as a more reliable cellular model for studying microglial biology and a promising cell source for *in vitro* models and cell transplantation (Gao et al., 2024). Furthermore, iMG can be generated with patient-specific microglial phenotypes carrying genetic mutations, allowing for the application of CRISPR gene-editing techniques to modify microglial phenotypes (Gao et al., 2024).

The co-culture models described above provide an optimized platform for retinal disease modeling and drug screening. They also facilitate deeper investigations into the mechanisms underlying retinal and central nervous system-related diseases, as well as the development of novel therapeutic strategies.

#### 4.1.2 Spontaneous generation of immune cells

In a study conducted by Shiraki et al. on hiPSCs, the researchers successfully expanded the cells into a self-formed ectodermal autonomous multi-zone (SEAM), which partially simulates human eye development. Unlike traditional microglial co-culture models that introduce microglia at later stages, this study revealed that microglia-like cells, exhibiting characteristics akin to yolk-sac lineage, could naturally develop within 2D SEAM ocular organoids, even without any vascular components (Shiraki et al., 2022). Similar instances of spontaneous microglia formation have been documented in brain organoid studies (Quadrato et al., 2017; Ormel et al., 2018; Sabate-Soler et al., 2022), with some researchers attributing this phenomenon to residual mesodermal progenitor cells. The signaling interactions between neural progenitor cells and mesodermal progenitor cells underscore the importance of localized cell-cell communication, potentially offering critical biological insights into microglial differentiation.

The spontaneous generation of microglia during organoid culture appears to be closely linked to their origins and developmental characteristics. Although SEAM ocular organoids lack vascular components, their 2D culture environment can still partially replicate embryonic developmental conditions, such as localized hypoxic environments, specific biochemical signaling molecules, and cell-cell interactions. Collectively, these factors may contribute to the differentiation of microglia-like cells. The formation of microglia is likely driven by intrinsic biological processes, where progenitor cells possess specific genetic and epigenetic regulatory mechanisms. Under organoid culture conditions, these mechanisms may become activated, facilitating the induction and differentiation of microglia.

These findings not only enhance our understanding of the origins of microglia but also provide valuable insights for developing more complex organoid models in the future, aimed at studying the interactions between microglia and neural cells.

### 4.2 Functional studies of immune cells in organoid models

Developing RO models that incorporate functional microglia has historically posed significant challenges in the field. Current research not only investigates the integration of individually cultured microglia into ROs but also emphasizes the importance of transplanted immune cells exhibiting in vivo-like activity.

Usui-Ouchi and colleagues made significant strides in creating RO models with mature and functional microglia. Their study revealed that by the sixth week of microglial RO co-culture, MPCs in the OPL displayed characteristics typical of mature microglia, such as small cell bodies and long branching processes extending along synapses. Further validation confirmed that these microglia expressed specific gene markers and proteins associated with mature microglia, indicating their functional maturation (Usui-Ouchi et al., 2023).

Chichagova et al. integrated iMG-like cells derived from hiPSCs into ROs, assessing their ability to migrate into the retina and express relevant functional markers. In their co-culture model, iMG cells responded to endotoxins in both monoculture and co-culture with ROs, as evidenced by a significant upregulation of pro-inflammatory markers, including IL-12/IL-23p40, IL-15, IL-16, TNF-β, IL-1β, IL-8, and TNF-α. Additionally, levels of the anti-inflammatory cytokine IL-10 and the anti-tumor mediator IL-13 were also found to be elevated (Chichagova et al., 2023). Previous studies (Laffer et al., 2019) have demonstrated that IL-10 possesses neuroprotective properties, mitigating inflammation-mediated neurodegeneration and reducing retinal microglial responses to lipoproteins. IL-13, an inflammatory regulator linked to uveitis (Roberge et al., 1998), has been shown to alleviate ocular inflammation in response to LPS(165). These findings underscore the capacity of microglia to maintain retinal homeostasis and illustrate that iMG-like cells retain essential functional properties (Chichagova et al., 2023).

Gao and colleagues discovered that co-culturing iMG with ROs enhances the functional maturation of iMG. Compared to iMG cultured alone, those in the co-culture model (CC-iMG) with ROs exhibited increased inward and outward rectifying K+ currents, along with a significant rise in intracellular lysosomes and mitochondria, indicating improved physiological functions such as phagocytic activity. To further explore the response of iMG to pro-inflammatory stimuli, the cells were exposed to a low concentration of bacterial endotoxin LPS (1 ng/mL) to simulate chronic inflammation. After 6 hours, iMG demonstrated successful activation, with significantly increased expression of CD68, MPO, and TNFα, sustained for 24 h. Additionally, to model the response of iMG to viral infection, the organoids were exposed to a high concentration of the TLR3 agonist dsRNA poly (I:C) (1 μg/mL). This exposure resulted in notable morphological changes in the microglia, with 1,458 genes downregulated and 465 genes upregulated, indicating a decrease in cellular activity. Graphene oxide analysis revealed enrichment in viral response and antiviral defense pathways, suggesting that poly (I:C) treatment elicited a robust pro-inflammatory response in iMG. These experiments demonstrate that iMG in the co-culture RO model exhibit in vivo-like functionality under both physiological and pathological conditions (Gao et al., 2024).

**FIGURE 3:**
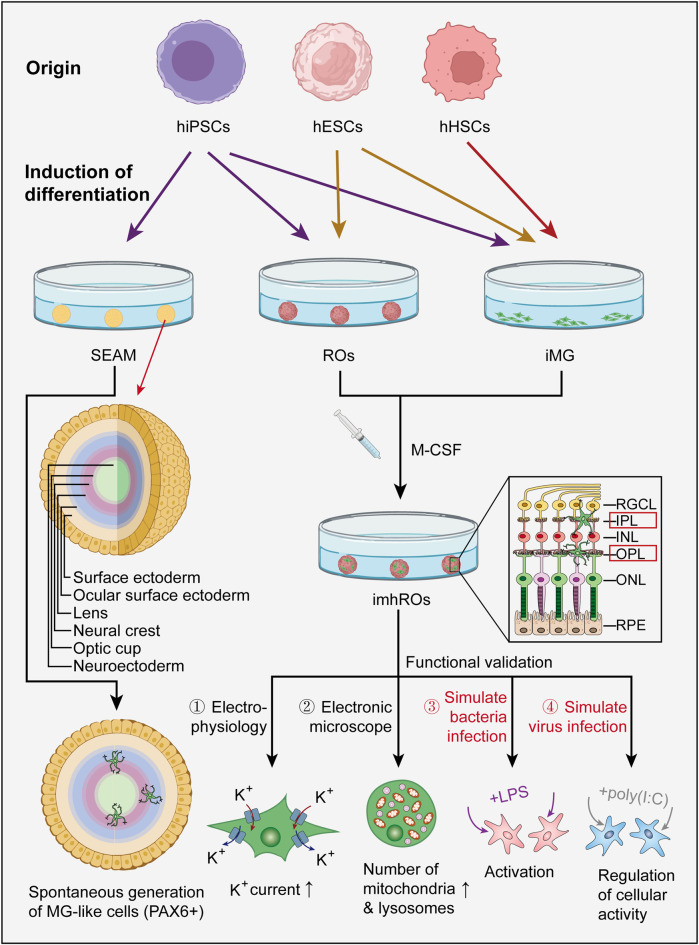
Current generation pathways of microglial ROs and the functional evaluation of iMG. Most existing studies generate microglial ROs through an additive approach, where hiPSCs or hESCs are first differentiated into iMG and ROs (iMG can also be derived from hHSCs), and then co-cultured together. SEAM is one case where microglia-like cells spontaneously form in ocular organoids. Functional evaluation of iMG in the co-culture model can be further performed using techniques such as electrophysiological recordings, electron microscopy observation, and by simulating bacterial or viral infections with LPS or poly (I:C). (hiPSC: human induced pluripotent stem cell, hESC: human embryonic stem cell, hHSC: human hematopoietic stem cell, SEAM: self-formed ectodermal autonomous multi-zone, iMG: induced microglia, M-CSF: macrophage colony stimulating factor, imhRO: immunized human retinal organoid, LPS: lipopolysaccharide).

While the functional characterization of co-cultured microglia remains incomplete and lacks systematic evaluation criteria, it is clear that current techniques have enabled the preliminary reconstruction of a relatively mature retinal microglial niche. These advancements have facilitated the induction of microglia with morphology and functionality resembling their *in vivo* counterparts. This progress has undoubtedly instilled confidence in researchers, paving the way for future efforts to harness the unique functions of microglia *in vitro* to model retinal diseases. Such advancements will establish a foundation for exploring the specific roles of the immune system in disease mechanisms and identifying new therapeutic targets (Figure 3).

## 5 Discussion

### 5.1 Technical limitations of Co-culturing ROs with immune cells

Although current ROs can replicate the structure and cellular composition of the retina, they still display notable differences in both structural and functional maturity when compared to adult retinal tissue. A significant limitation is the inability of RGCs to survive in substantial numbers during the later stages of RO maturation, resulting in an almost complete absence of the IPL (166). In contrast, the IPL and RGCs are essential components of the microglial niche within the retina (Schmied et al., 2024). Enhancing the survival of the inner retinal layers is crucial for the effective integration of microglia. Furthermore, the process of injecting differentiated microglia into organoids can inflict physical damage on the existing cellular structure (Bhaduri et al., 2020; Boisvert et al., 2019; Paşca et al., 2019). Minimizing this disruption to the original culture presents a practical challenge that must be addressed to improve co-culture models.

Another critical limitation is the relatively low number of microglia integrated into ROs, which fails to achieve saturation of microglial populations within the OPL (69). Given the inherently low proliferation rate of microglia, extending the culture period is unlikely to yield a larger population. Beyond sheer population size, the diversity of microglia in current microglial RO models is also restricted. In the actual *in vivo* environment of retinal diseases, distinct microglial subpopulations may exist in various states, each with specific functions. Animal models have demonstrated that certain microglial subtypes are linked to conditions such as oxygen-induced retinopathy or light-induced photoreceptor degeneration (O'Koren et al., 2019; He et al., 2021). Increasing the diversity and heterogeneity of immune cells could facilitate the identification of therapeutic targets; however, achieving this level of complexity presents a significant technical challenge.

In addition to the resident immune cells within the retina, external immune cells, such as B cells and T cells, may also play a role in the onset and progression of retinal diseases when immune privilege is compromised (He et al., 2024). Nevertheless, most current ROs lack a mature vascular system and dynamic fluid conditions, complicating the modeling of the pathological processes involved in recruiting immune cells from outside the retina during inflammatory responses. This limitation somewhat diminishes the clinical translational value of co-culture models.

Regarding cultivation strategies and experimental parameters, research on co-culturing ROs with immune cells suffers from a lack of standardized protocols. Experimental designs, co-culture durations, immune cell ratios, and evaluation metrics vary widely without uniform guidelines. Additionally, the functional assessment of immune cells remains relatively simplistic, making it challenging to ascertain whether the responses of *in vitro* models to external stimuli can accurately replicate the mechanisms observed *in vivo* (Table 1).

**TABLE 1:** Differences between traditional and microglial-integrated ROs.

Model	Cell type	Culture method	Advantages	Limitations
Traditional ROs	Photoreceptors (rods and cones), RGC, bipolar cells, horizontal cells, astrocytes, Müller glia, RPE	Induction of stem cell differentiation	1. Well-established and highly standardized procedure 2. Easy to replicate and scale up for mass production 3. Fundamental research on retinal development and differentiation	1. Lack of the immune system, leading to an incomplete model of retinal diseases 2. Lack of the vascular system, preventing long-term survival of cells in deeper regions 3. Gaps in tissue structure and cellular function compared to *in vivo* retina
Microglial-integrated ROs	All of the cell types contained in traditional ROs + microglia	1. Ectopic addition of microglia 2. Spontaneous differentiation of microglia	1. Retinal disease modeling by simulating immune responses 2. Simulation of immunological rejection between the host and the graft 3. Promotion of differentiation and maturation of other retinal cells 4. Immunomodulatory drug screening and immunotherapy safety evaluation	1. Limited methods for inducing the spontaneous differentiation of microglia 2. Lack of standardized protocols and functional identification 3. Limited number and subtypes of microglia in co-culture systems 4. Absence of an integrated vascular system

### 5.2 Challenges and prospects

Future research should prioritize the optimization of co-culture conditions to enhance the long-term survival of iMG. Potential strategies may include refining co-culture media, integrating microfluidic platforms, and employing organ-on-chip technologies.

Optimizing culture medium parameters entails adjusting nutrient composition, the ratio of organoid to immune cells, and incorporating essential cytokines that facilitate immune cell migration and development. Furthermore, the integration of microfluidic devices can significantly improve the distribution of oxygen and nutrients, thereby enhancing the overall culture environment (Gong et al., 2023).

Microfluidic systems typically provide compartmentalized setups that support the co-culture of various cell types. These compartments, separated by semi-permeable support membranes, allow for the formation of monolayers of cultured cells. Over the past decade, the integration of advanced microfluidic systems with organoids has transformed and broadened their applications, particularly in medical and pharmaceutical research (Ingber, 2022; Low et al., 2021; Bhatia and Ingber, 2014). The use of microfluidics has improved system output and increased the complexity of cell assembly, enabling the simulation of one or multiple organs and facilitating the study of intra- and inter-organ signaling. As the number of compartments increases, microfluidic systems can accommodate more cell types and physiological conditions, further enhancing the system’s complexity (Bein et al., 2018; Takebe et al., 2017; Wu et al., 2020).

While organoids have effectively replicated the local microenvironment of the retina, integrating the retinal immune system with the systemic immune system necessitates more sophisticated technologies, such as “multi-organ-on-chip” systems. Organ-on-chip technology, as described in numerous studies, is currently available on a limited number of commercial platforms (Achberger et al., 2019; Carvalho et al., 2023). This technology involves culturing human cells, tissues, or organoids on miniaturized platforms to mimic the physiological and pathological characteristics of human organs. The combination of organ-on-chip technology with microfluidics and organoid systems presents significant advantages for studying cellular responses to drugs, immune cells, or microbial compounds (Görgens et al., 2021; Šuligoj et al., 2020). High-throughput screening techniques (Naumovska et al., 2020; Beaurivage et al., 2019) can be employed to investigate interactions between identical cell types and cells from different tissues, signaling compounds, or drugs (Wevers et al., 2018; Spijkers et al., 2021; Gijzen et al., 2020; Maisonneuve et al., 2021). Conversely, these systems can also be utilized to assess the responses of cells from various organoid systems or donors under identical external stimuli, allowing for the detection of patient-specific differences and advancing personalized medicine.

Previous studies have indicated that co-culturing iMG with astrocytes or neurons can enhance iMG maturation (Abud et al., 2017). Similarly, iPSC-derived microglia have been shown to promote the maturation of cerebral organoids (COs) through cholesterol transfer (Park et al., 2023). Therefore, exploring the interactions between retinal neurons and microglia is essential for understanding microglial biology. Research on co-culturing microglia with COs predates similar studies with ROs, providing valuable insights into organoid cultivation methods, immune cell integration, and the construction of in vivo-like microenvironments. As the resident immune cells of the brain, microglia constitute approximately 5%–10% of the total cells in the CNS(191), coordinating brain inflammatory responses (Salter and Beggs, 2014) and forming the immune microenvironment alongside astrocytes (Ao et al., 2021). For instance, Sun et al. developed COs with vascular networks and microglia capable of activation under immune stimulation, even simulating functional BBB-like structures. This model serves as a platform for studying interactions between neuronal and non-neuronal components during brain development (Sun et al., 2022). Park et al. created a co-culture system of neurons, astrocytes, and microglia to investigate their interactions in an AD model (Park et al., 2018). Lin et al. advanced this approach by co-culturing iPSC-derived microglia carrying AD-related APOE4 mutations with COs to study their uptake of amyloid β-protein (Lin et al., 2018). To simulate inflammation, Dos et al. transplanted infected microglia into COs for co-culture (Dos Reis et al., 2020), while Narasipura’s team demonstrated that hematopoietic progenitor cells could integrate into mature COs, proliferate, and differentiate into microglia, achieving gradual maturation. In this model, microglia accounted for approximately 7.3% of the total cells, compared to less than 1% in most conventional COs. Following HIV infection, the microglial CO model exhibited enhanced neuroinflammatory characteristics (Narasipura et al., 2024). Ao et al. introduced an innovative tubular CO model by integrating 3D-printed hollow lattice scaffolds into porous plates, enabling scalable, renewable, and reliable tubular organoids. This tubular structure can deliver nutrients, oxygen, and immune cells, facilitating non-invasive immune integration, improving organoid culture, and simulating neuro-immune interactions (Ao et al., 2021). The successful experience with microglial COs can serve as a valuable guide for developing more advanced and comprehensive microglial RO models. By replicating increasingly complex systems and leveraging multi-system synergistic support, these models can facilitate the co-development of immune cells and neural cells more effectively.

Microglia are increasingly recognized as critical targets for discovering new drugs or therapies. Strategies under exploration include reprogramming microglia using homeostatic signals or other small molecules, depleting microglia with chemical agents or radiation, and inhibiting downstream microglial functions by blocking cytokine activity or phagocytosis (Wang and Cepko, 2022). Simultaneously, microglia can also play beneficial roles in ocular diseases. Some animal model studies have unexpectedly revealed the protective effects of microglia in retinal degeneration (Wang et al., 2021). Similarly, research in neuroscience has demonstrated that microglia positively regulate the microenvironment in injured brains and neurodegenerative diseases such as AD (200). Consequently, microglial replacement therapy is emerging as a promising therapeutic approach. Additionally, microglial genes exhibit regional diversity, and future work could leverage genetic and epigenetic tools to generate retina-specific iMG. For disease modeling, iPSCs derived from specific patient populations or ROs cultured under pathological conditions could be developed into human retinal disease models, providing valuable platforms to study disease mechanisms and support future drug discovery efforts. By continually advancing the development of microglial ROs that better replicate the *in vivo* microenvironment, we can gain more meaningful insights into the roles and functions of immune cells in retinal pathology.

In developing more complex system models, microglia play a unique role in vascular formation under both physiological and pathological conditions in the retina (Simmons et al., 2016). Studies have shown that LPS-activated microglia can promote angiogenesis, migration, proliferation, and increased permeability of co-cultured retinal microvascular endothelial cells (Ding et al., 2018). The vascular system serves as a natural mechanism for immune responses, as traditional immunotherapies heavily rely on vascular transport. A functional vascular network allows immune cells and signaling molecules to reach sites of injury or tumorigenesis, facilitating effective immune responses. Thus, the development of comprehensive microglial ROs largely depends on the incorporation of a vascular system (Ehlers et al., 2023; Wu et al., 2021). This not only enhances the recruitment of immune cells and improves the efficiency of immune responses but also enables the simulation of the most realistic physiological and pathological processes in a multi-system environment closely resembling *in vivo* tissue structures (Shin et al., 2021).

From a localized perspective to a broader view, while 3D-RO models have been successfully developed (Eiraku et al., 2011; Zhong et al., 2014), a complete eyeball 3D organoid model—encompassing the cornea, conjunctiva, and lens at the front to the retina and choroid at the back—has yet to be successfully created. Developing 3D models of the human eyeball would provide valuable insights into the origins and mechanisms of the immune system’s functions. Such advancements in methods and technologies will rely on the support of new infrastructures, such as organoid biobanks. The future holds significant potential for further exploration in this area.

## 6 Conclusion

To create *in vitro* models that more accurately mimic the human retinal microenvironment, it is crucial to continuously diversify the range of cell types incorporated into ROs. This article emphasizes the eye’s distinctive “immune privilege” mechanism, detailing the composition of immune cells in both physiological and pathological states, and their relationship with retinal diseases, highlighting the immune system’s significance. By integrating immune cells into organoids, researchers can achieve more precise disease modeling, promote therapeutic research, and enhance the prospects for clinical application.

Given the current limitations in stem cell induction technologies, most co-culture RO models still depend on processing “seed cells” through various pathways, followed by the integration of independently induced ROs and microglia. Although some studies have indicated that microglia can spontaneously develop in ocular organoids, the underlying reasons for this occurrence remain unclear. Future investigations should focus more on uncovering the origins of microglia in ROs, as this could be a pivotal strategy for overcoming the challenges associated with creating organoid models that feature more intricate systems.

In conclusion, microglial ROs act as a vital link between fundamental research and clinical application. They not only provide researchers with fresh insights into retinal immune functions and disease mechanisms but also present extensive opportunities for drug development and personalized therapies. With continuous technological advancements, microglial ROs are set to assume an increasingly significant role in future ophthalmic research, paving the way for new breakthroughs in the treatment of retinal diseases.

## Glossary

(RO): retinal organoid
(MHCII): major histocompatibility complex II
(aSC): adult stem cell
(PSC): pluripotent stem cell
(ePSC): embryonic pluripotent stem cell
(iPSC): induced pluripotent stem cell
(RPC): retinal progenitor cell
(RGC): retinal ganglion cell
(RPE): retinal pigment epithelium
(BRB): blood-retinal barrier
(APC): antigen-presenting cell
(CNS): central nervous system
(BBB): blood-brain barrier
(IPL): inner plexiform layer
(OPL): outer plexiform layer
(ONL): outer nuclear layer
(HSC): hematopoietic stem cell
(DR): diabetic retinopathy
(RB): retinoblastoma
(NK cell): natural killer cell
(AMD): age-related macular degeneration
(RP): retinitis pigmentosa
(RVO): retinal vein occlusion
(CNV): choroidal neovascularization
(AD): Alzheimer's Disease
(NMDA): Nmethyl-D-aspartate
(MEM): memantine
(LPS): lipopolysaccharide
(VEGF): vascular endothelial growth factor
(hESC): human embryonic stem cell
(MPC): macrophage precursor cell
(hiPSC): human induced pluripotent stem cell
(iMG): induced microglia
(hHSC): human hematopoietic stem cell
(SEAM): self-formed ectodermal autonomous multi-zone
(CO): cerebral organoid

## References

[B1] Abud E. M. Ramirez R. N. Martinez E. S. Healy L. M. Nguyen C. H. H. Newman S. A. (2017). iPSC-derived human microglia-like cells to study neurological diseases. Neuron 94 (2), 278–293. 10.1016/j.neuron.2017.03.042 28426964 PMC5482419

[B2] Achberger K. Probst C. Haderspeck J. Bolz S. Rogal J. Chuchuy J. (2019). Merging organoid and organ-on-a-chip technology to generate complex multi-layer tissue models in a human retina-on-a-chip platform. Elife 8, e46188. 10.7554/eLife.46188 31451149 PMC6777939

[B3] Aires I. D. Ribeiro-Rodrigues T. Boia R. Catarino S. Girão H. Ambrósio A. F. (2020). Exosomes derived from microglia exposed to elevated pressure amplify the neuroinflammatory response in retinal cells. Glia 68 (12), 2705–2724. 10.1002/glia.23880 32645245

[B4] Akhtar T. Xie H. Khan M. I. Zhao H. Bao J. Zhang M. (2019). Accelerated photoreceptor differentiation of hiPSC-derived retinal organoids by contact co-culture with retinal pigment epithelium. Stem Cell Res. 39, 101491. 10.1016/j.scr.2019.101491 31326746

[B5] Andrews M. G. Kriegstein A. R. (2022). Challenges of organoid research. Annu. Rev. Neurosci. 45, 23–39. 10.1146/annurev-neuro-111020-090812 34985918 PMC10559943

[B6] Ao Z. Cai H. Wu Z. Song S. Karahan H. Kim B. (2021). Tubular human brain organoids to model microglia-mediated neuroinflammation. Lab. Chip 21 (14), 2751–2762. 10.1039/d1lc00030f 34021557 PMC8493632

[B7] Araya-Arriagada J. Bello F. Shivashankar G. Neira D. Durán-Aniotz C. Acosta M. L. (2021). Retinal ganglion cells functional changes in a mouse model of Alzheimer's disease are linked with neurotransmitter alterations. J. Alzheimers Dis. 82 (s1), S5–s18. 10.3233/JAD-201195 33749647

[B8] Azzam R. M. Yehia S. Saleh A. Mohamed A. Tohamy D. M. (2024). Visual outcomes of traumatic posterior segment complications in upper Egypt: tertiary center study. J. Curr Med. Res. Pract. 10.21608/jcmrp.2024.346800

[B9] Bamforth S. D. Lightman S. L. Greenwood J. (1997). Interleukin-1 beta-induced disruption of the retinal vascular barrier of the central nervous system is mediated through leukocyte recruitment and histamine. Am. J. Pathol. 150 (1), 329–340. 9006348 PMC1858506

[B10] Barresi C. Chhablani J. Dolz-Marco R. Gallego-Pinazo R. Berni A. Bandello F. (2024). Retinal neurodegeneration in age-related macular degeneration. Eur. J. Ophthalmol. 34 (3), 624–630. 10.1177/11206721231186166 37394731

[B11] Beaurivage C. Naumovska E. Chang Y. X. Elstak E. D. Nicolas A. Wouters H. (2019). Development of a gut-on-A-chip model for high throughput disease modeling and drug discovery. Int. J. Mol. Sci. 20 (22), 5661. 10.3390/ijms20225661 31726729 PMC6888156

[B12] Bein A. Shin W. Jalili-Firoozinezhad S. Park M. H. Sontheimer-Phelps A. Tovaglieri A. (2018). Microfluidic organ-on-a-chip models of human intestine. Cell Mol. Gastroenterol. Hepatol. 5 (4), 659–668. 10.1016/j.jcmgh.2017.12.010 29713674 PMC5924739

[B13] Bell C. M. Zack D. J. Berlinicke C. A. (2020). Human organoids for the study of retinal development and disease. Annu. Rev. Vis. Sci. 6, 91–114. 10.1146/annurev-vision-121219-081855 32936736

[B14] Benhar I. Reemst K. Kalchenko V. Schwartz M. (2016). The retinal pigment epithelium as a gateway for monocyte trafficking into the eye. Embo J. 35 (11), 1219–1235. 10.15252/embj.201694202 27107049 PMC4888238

[B15] Beryozkin A. Khateb S. Idrobo-Robalino C. A. Khan M. I. Cremers F. P. M. Obolensky A. (2020). Unique combination of clinical features in a large cohort of 100 patients with retinitis pigmentosa caused by FAM161A mutations. Sci. Rep. 10 (1), 15156. 10.1038/s41598-020-72028-0 32938956 PMC7495424

[B16] Bhaduri A. Andrews M. G. Mancia Leon W. Jung D. Shin D. Allen D. (2020). Cell stress in cortical organoids impairs molecular subtype specification. Nature 578 (7793), 142–148. 10.1038/s41586-020-1962-0 31996853 PMC7433012

[B17] Bhatia S. N. Ingber D. E. (2014). Microfluidic organs-on-chips. Nat. Biotechnol. 32 (8), 760–772. 10.1038/nbt.2989 25093883

[B18] Boisvert E. M. Means R. E. Michaud M. Madri J. A. Katz S. G. (2019). Minocycline mitigates the effect of neonatal hypoxic insult on human brain organoids. Cell Death Dis. 10 (4), 325. 10.1038/s41419-019-1553-x 30975982 PMC6459920

[B19] Bravo-Gil N. González-Del Pozo M. Martín-Sánchez M. Méndez-Vidal C. Rodríguez-de la Rúa E. Borrego S. (2017). Unravelling the genetic basis of simplex Retinitis Pigmentosa cases. Sci. Rep. 7, 41937. 10.1038/srep41937 28157192 PMC5291209

[B20] Brooks M. J. Chen H. Y. Kelley R. A. Mondal A. K. Nagashima K. De V. N. (2019). Improved retinal organoid differentiation by modulating signaling pathways revealed by comparative transcriptome analyses with development *in vivo* . Stem Cell Rep. 13 (5), 891–905. 10.1016/j.stemcr.2019.09.009 PMC6895716 31631019

[B21] Brown G. C. Neher J. J. (2014). Microglial phagocytosis of live neurons. Nat. Rev. Neurosci. 15 (4), 209–216. 10.1038/nrn3710 24646669

[B22] Cai L. Xia M. Zhang F. (2024). Redox regulation of immunometabolism in microglia underpinning diabetic retinopathy. Antioxidants (Basel) 13 (4), 423. 10.3390/antiox13040423 38671871 PMC11047590

[B23] Cameron O. Neves J. F. Gentleman E. (2024). Listen to your gut: key concepts for bioengineering advanced models of the intestine. Adv. Sci. (Weinh) 11 (5), e2302165. 10.1002/advs.202302165 38009508 PMC10837392

[B24] Carvalho M. R. Yan L. P. Li B. Zhang C. H. He Y. L. Reis R. L. (2023). Gastrointestinal organs and organoids-on-a-chip: advances and translation into the clinics. Biofabrication 15 (4), 042004. 10.1088/1758-5090/acf8fb 37699408

[B25] Casamitjana J. Espinet E. Rovira M. (2022). Pancreatic organoids for regenerative medicine and cancer research. Front. Cell Dev. Biol. 10, 886153. 10.3389/fcell.2022.886153 35592251 PMC9110799

[B26] Castillo García M. Urdapilleta E. (2022). Dynamical adaptation in photoreceptors with gain control. Phys. Biol. 19 (6), 066006. 10.1088/1478-3975/ac9947 36220008

[B27] Chakrabarti J. Koh V. So J. B. Y. Yong W. P. Zavros Y. (2021). A preclinical human-derived autologous gastric cancer organoid/immune cell Co-culture model to predict the efficacy of targeted therapies. J. Vis. Exp. (173). 10.3791/61443 34309588

[B28] Chang K. C. Shieh B. Petrash J. M. (2019). Role of aldose reductase in diabetes-induced retinal microglia activation. Chem. Biol. Interact. 302, 46–52. 10.1016/j.cbi.2019.01.020 30682331 PMC6421082

[B29] Checchin D. Sennlaub F. Levavasseur E. Leduc M. Chemtob S. (2006). Potential role of microglia in retinal blood vessel formation. Invest Ophthalmol. Vis. Sci. 47 (8), 3595–3602. 10.1167/iovs.05-1522 16877434

[B30] Chen J. Lu J. Wang S. N. Miao C. Y. (2024). Application and challenge of pancreatic organoids in therapeutic research. Front. Pharmacol. 15, 1366417. 10.3389/fphar.2024.1366417 38855754 PMC11157021

[B31] Chen Y. Xia Q. Zeng Y. Zhang Y. Zhang M. (2022). Regulations of retinal inflammation: focusing on müller glia. Front. Cell Dev. Biol. 10, 898652. 10.3389/fcell.2022.898652 35573676 PMC9091449

[B32] Cherne M. D. Sidar B. Sebrell T. A. Sanchez H. S. Heaton K. Kassama F. J. (2021). A synthetic hydrogel, VitroGel(®) ORGANOID-3, improves immune cell-epithelial interactions in a tissue chip Co-culture model of human gastric organoids and dendritic cells. Front. Pharmacol. 12, 707891. 10.3389/fphar.2021.707891 34552484 PMC8450338

[B33] Cherry J. D. Olschowka J. A. O'Banion M. K. (2014). Neuroinflammation and M2 microglia: the good, the bad, and the inflamed. J. Neuroinflammation 11, 98. 10.1186/1742-2094-11-98 24889886 PMC4060849

[B34] Chew S. H. Martinez C. Chirco K. R. Kandoi S. Lamba D. A. (2022). Timed notch inhibition drives photoreceptor fate specification in human retinal organoids. Invest Ophthalmol. Vis. Sci. 63 (10), 12. 10.1167/iovs.63.10.12 PMC9513742 36129723

[B35] Chichagova V. Georgiou M. Carter M. Dorgau B. Hilgen G. Collin J. (2023). Incorporating microglia‐like cells in human induced pluripotent stem cell‐derived retinal organoids. J. Cell. Mol. Med. 27 (3), 435–445. 10.1111/jcmm.17670 36644817 PMC9889627

[B36] Choi W. H. Bae D. H. Yoo J. (2023). Current status and prospects of organoid-based regenerative medicine. BMB Rep. 56 (1), 10–14. 10.5483/BMBRep.2022-0195 36523211 PMC9887105

[B37] Colonna M. Butovsky O. (2017). Microglia function in the central nervous system during health and neurodegeneration. Annu. Rev. Immunol. 35, 441–468. 10.1146/annurev-immunol-051116-052358 28226226 PMC8167938

[B38] Couturier A. Bousquet E. Zhao M. Naud M. C. Klein C. Jonet L. (2014). Anti-vascular endothelial growth factor acts on retinal microglia/macrophage activation in a rat model of ocular inflammation. Mol. Vis. 20, 908–920. 24966662 PMC4067232

[B39] Cowan C. S. Renner M. De Gennaro M. Gross-Scherf B. Goldblum D. Hou Y. (2020). Cell types of the human retina and its organoids at single-cell resolution. Cell 182 (6), 1623–1640. 10.1016/j.cell.2020.08.013 32946783 PMC7505495

[B40] Cruz-Gálvez C. C. Ordaz-Favila J. C. Villar-Calvo V. M. Cancino-Marentes M. E. Bosch-Canto V. (2022). Retinoblastoma: review and new insights. Front. Oncol. 12, 963780. 10.3389/fonc.2022.963780 36408154 PMC9670800

[B41] Cuevas E. Holder D. L. Alshehri A. H. Tréguier J. Lakowski J. Sowden J. C. (2021). NRL(-/-) gene edited human embryonic stem cells generate rod-deficient retinal organoids enriched in S-cone-like photoreceptors. Stem Cells 39 (4), 414–428. 10.1002/stem.3325 33400844 PMC8438615

[B42] Ding X. Gu R. Zhang M. Ren H. Shu Q. Xu G. (2018). Microglia enhanced the angiogenesis, migration and proliferation of co-cultured RMECs. BMC Ophthalmol. 18 (1), 249. 10.1186/s12886-018-0886-z 30223824 PMC6142340

[B43] Dorgau B. Georgiou M. Chaudhary A. Moya-Molina M. Collin J. Queen R. (2022). Human retinal organoids provide a suitable tool for toxicological investigations: a comprehensive validation using drugs and compounds affecting the retina. Stem Cells Transl. Med. 11 (2), 159–177. 10.1093/stcltm/szab010 35298655 PMC8929478

[B44] Dos Reis R. S. Sant S. Keeney H. Wagner M. C. E. Ayyavoo V. (2020). Modeling HIV-1 neuropathogenesis using three-dimensional human brain organoids (hBORGs) with HIV-1 infected microglia. Sci. Rep. 10 (1), 15209. 10.1038/s41598-020-72214-0 32938988 PMC7494890

[B45] Ebneter A. Kokona D. Schneider N. Zinkernagel M. S. (2017). Microglia activation and recruitment of circulating macrophages during ischemic experimental branch retinal vein occlusion. Invest Ophthalmol. Vis. Sci. 58 (2), 944–953. 10.1167/iovs.16-20474 28170538

[B46] Edler M. K. Mhatre-Winters I. Richardson J. R. (2021). Microglia in aging and Alzheimer's disease: a comparative species review. Cells. 10 (5), 1138. 10.3390/cells10051138 34066847 PMC8150617

[B47] Ehlers H. Nicolas A. Schavemaker F. Heijmans J. P. M. Bulst M. Trietsch S. J. (2023). Vascular inflammation on a chip: a scalable platform for trans-endothelial electrical resistance and immune cell migration. Front. Immunol. 14, 1118624. 10.3389/fimmu.2023.1118624 36761747 PMC9903066

[B48] Eiraku M. Takata N. Ishibashi H. Kawada M. Sakakura E. Okuda S. (2011). Self-organizing optic-cup morphogenesis in three-dimensional culture. Nature 472 (7341), 51–56. 10.1038/nature09941 21475194

[B49] Fan W. Huang W. Chen J. Li N. Mao L. Hou S. (2022). Retinal microglia: functions and diseases. Immunology 166 (3), 268–286. 10.1111/imm.13479 35403700

[B50] Fang H. Xu H. Yu J. Cao H. Li L. (2024). Human hepatobiliary organoids: recent advances in drug toxicity verification and drug screening. Biomolecules 14 (7), 794. 10.3390/biom14070794 39062508 PMC11274902

[B51] Fathi M. Ross C. T. Hosseinzadeh Z. (2021). Functional 3-dimensional retinal organoids: technological progress and existing challenges. Front. Neurosci. 15, 668857. 10.3389/fnins.2021.668857 33958988 PMC8095320

[B52] Fige É. Sarang Z. Sós L. Szondy Z. (2022). Retinoids promote mouse bone marrow-derived macrophage differentiation and efferocytosis via upregulating bone morphogenetic protein-2 and Smad3. Cells 11 (18), 2928. 10.3390/cells11182928 36139503 PMC9497139

[B53] Fletcher E. L. (2020). Contribution of microglia and monocytes to the development and progression of age related macular degeneration. Ophthalmic Physiol. Opt. 40 (2), 128–139. 10.1111/opo.12671 32017190

[B54] Frost J. L. Schafer D. P. (2016). Microglia: architects of the developing nervous system. Trends Cell Biol. 26 (8), 587–597. 10.1016/j.tcb.2016.02.006 27004698 PMC4961529

[B55] Gallenga C. E. Lonardi M. Pacetti S. Violanti S. S. Tassinari P. Di Virgilio F. (2021). Molecular mechanisms related to oxidative stress in retinitis pigmentosa. Antioxidants (Basel) 10 (6), 848. 10.3390/antiox10060848 34073310 PMC8229325

[B56] Ganzen L. Yadav S. C. Wei M. Ma H. Nawy S. Kramer R. H. (2024). Retinoic acid-dependent loss of synaptic output from bipolar cells impairs visual information processing in inherited retinal degeneration. J. Neurosci. 44 (35), e0129242024. 10.1523/JNEUROSCI.0129-24.2024 39060177 PMC11358532

[B57] Gao H. A L. Huang X. Chen X. Xu H. (2021). Müller glia-mediated retinal regeneration. Mol. Neurobiol. 58 (5), 2342–2361. 10.1007/s12035-020-02274-w 33417229

[B58] Gao L. Chen X. Tang Y. Zhao J. Li Q. Fan X. (2015). Neuroprotective effect of memantine on the retinal ganglion cells of APPswe/PS1ΔE9 mice and its immunomodulatory mechanisms. Exp. Eye Res. 135, 47–58. 10.1016/j.exer.2015.04.013 25912193

[B59] Gao M.-L. Wang T.-Y. Lin X. Tang C. Li M. Bai Z.-P. (2024). Retinal organoid microenvironment enhanced bioactivities of microglia-like cells derived from HiPSCs. Investigative Ophthalmol. and Vis. Sci. 65 (12), 19. 10.1167/iovs.65.12.19 PMC11472886 39392440

[B60] Gijzen L. Marescotti D. Raineri E. Nicolas A. Lanz H. L. Guerrera D. (2020). An intestine-on-a-chip model of plug-and-play modularity to study inflammatory processes. SLAS Technol. 25 (6), 585–597. 10.1177/2472630320924999 32576063 PMC7684793

[B61] Ginhoux F. Greter M. Leboeuf M. Nandi S. See P. Gokhan S. (2010). Fate mapping analysis reveals that adult microglia derive from primitive macrophages. Science 330 (6005), 841–845. 10.1126/science.1194637 20966214 PMC3719181

[B62] Gong J. Gong Y. Zou T. Zeng Y. Yang C. Mo L. (2023). A controllable perfusion microfluidic chip for facilitating the development of retinal ganglion cells in human retinal organoids. Lab. Chip 23 (17), 3820–3836. 10.1039/d3lc00054k 37496497

[B63] Görgens C. Ramme A. P. Guddat S. Schrader Y. Winter A. Dehne E. M. (2021). Organ-on-a-chip: determine feasibility of a human liver microphysiological model to assess long-term steroid metabolites in sports drug testing. Drug Test. Anal. 13 (11-12), 1921–1928. 10.1002/dta.3161 34505743

[B64] Gosselin D. Skola D. Coufal N. G. Holtman I. R. Schlachetzki J. C. M. Sajti E. (2017). An environment-dependent transcriptional network specifies human microglia identity. Science 356 (6344), eaal3222. 10.1126/science.aal3222 28546318 PMC5858585

[B65] Grassi L. Alfonsi R. Francescangeli F. Signore M. De Angelis M. L. Addario A. (2019). Organoids as a new model for improving regenerative medicine and cancer personalized therapy in renal diseases. Cell Death Dis. 10 (3), 201. 10.1038/s41419-019-1453-0 30814510 PMC6393468

[B66] Guo L. Choi S. Bikkannavar P. Cordeiro M. F. (2022). Microglia: key players in retinal ageing and neurodegeneration. Front. Cell Neurosci. 16, 804782. 10.3389/fncel.2022.804782 35370560 PMC8968040

[B67] Gupta N. Brown K. E. Milam A. H. (2003). Activated microglia in human retinitis pigmentosa, late-onset retinal degeneration, and age-related macular degeneration. Exp. Eye Res. 76 (4), 463–471. 10.1016/s0014-4835(02)00332-9 12634111

[B68] Hansen D. V. Hanson J. E. Sheng M. (2018). Microglia in Alzheimer's disease. J. Cell Biol. 217 (2), 459–472. 10.1083/jcb.201709069 29196460 PMC5800817

[B69] Hautefort I. Poletti M. Papp D. Korcsmaros T. (2022). Everything you always wanted to know about organoid-based models (and never dared to ask). Cell Mol. Gastroenterol. Hepatol. 14 (2), 311–331. 10.1016/j.jcmgh.2022.04.012 35643188 PMC9233279

[B70] He C. Liu Y. Huang Z. Yang Z. Zhou T. Liu S. (2021). A specific RIP3(+) subpopulation of microglia promotes retinopathy through a hypoxia-triggered necroptotic mechanism. Proc. Natl. Acad. Sci. U. S. A. 118 (11), e2023290118. 10.1073/pnas.2023290118 33836603 PMC7980367

[B71] He S. Liu C. Ren C. Zhao H. Zhang X. (2024). Immunological landscape of retinal ischemia-reperfusion injury: insights into resident and peripheral immune cell responses. Aging Dis. 16, 115–136. 10.14336/AD.2024.0129 38502592 PMC11745425

[B72] Hoeffel G. Ginhoux F. (2018). Fetal monocytes and the origins of tissue-resident macrophages. Cell Immunol. 330, 5–15. 10.1016/j.cellimm.2018.01.001 29475558

[B73] Huang J. Wang X. Li N. Fan W. Li X. Zhou Q. (2024). YY1 lactylation aggravates autoimmune uveitis by enhancing microglial functions via inflammatory genes. Adv. Sci. (Weinh) 11 (19), e2308031. 10.1002/advs.202308031 38493498 PMC11109619

[B74] Hussey K. A. Hadyniak S. E. Johnston R. J. Jr (2022). Patterning and development of photoreceptors in the human retina. Front. Cell Dev. Biol. 10, 878350. 10.3389/fcell.2022.878350 35493094 PMC9049932

[B75] Ichinose T. Habib S. (2022). ON and OFF signaling pathways in the retina and the visual system. Front. Ophthalmol. (Lausanne) 2, 989002. 10.3389/fopht.2022.989002 36926308 PMC10016624

[B76] Ingber D. E. (2022). Human organs-on-chips for disease modelling, drug development and personalized medicine. Nat. Rev. Genet. 23 (8), 467–491. 10.1038/s41576-022-00466-9 35338360 PMC8951665

[B77] Jia Y. Xue W. Tong X. Wang Y. Cui L. Zou H. (2021). Quantitative analysis and clinical application of iris circulation in ischemic retinal disease. BMC Ophthalmol. 21 (1), 393. 10.1186/s12886-021-02165-1 34781913 PMC8594238

[B78] Jin N. Gao L. Fan X. Xu H. (2017). Friend or foe? Resident microglia vs bone marrow-derived microglia and their roles in the retinal degeneration. Mol. Neurobiol. 54 (6), 4094–4112. 10.1007/s12035-016-9960-9 27318678

[B79] Jin N. Sha W. Gao L. (2021). Shaping the microglia in retinal degenerative diseases using stem cell therapy: practice and prospects. Front. Cell Dev. Biol. 9, 741368. 10.3389/fcell.2021.741368 34966736 PMC8710684

[B80] Joo H. Min S. Cho S. W. (2024). Advanced lung organoids for respiratory system and pulmonary disease modeling. J. Tissue Eng. 15, 20417314241232502. 10.1177/20417314241232502 38406820 PMC10894554

[B81] Jowett G. M. Coales I. Neves J. F. (2022). Organoids as a tool for understanding immune-mediated intestinal regeneration and development. Development 149 (8), dev199904. 10.1242/dev.199904 35502785

[B82] Karlstetter M. Scholz R. Rutar M. Wong W. T. Provis J. M. Langmann T. (2015). Retinal microglia: just bystander or target for therapy? Prog. Retin Eye Res. 45, 30–57. 10.1016/j.preteyeres.2014.11.004 25476242

[B83] Keino H. Horie S. Sugita S. (2018). Immune privilege and eye-derived T-regulatory cells. J. Immunol. Res. 2018, 1679197. 10.1155/2018/1679197 29888291 PMC5985108

[B84] Kierdorf K. Erny D. Goldmann T. Sander V. Schulz C. Perdiguero E. G. (2013). Microglia emerge from erythromyeloid precursors via Pu.1- and Irf8-dependent pathways. Nat. Neurosci. 16 (3), 273–280. 10.1038/nn.3318 23334579

[B85] Kim S. Lowe A. Dharmat R. Lee S. Owen L. A. Wang J. (2019). Generation, transcriptome profiling, and functional validation of cone-rich human retinal organoids. Proc. Natl. Acad. Sci. U. S. A. 116 (22), 10824–10833. 10.1073/pnas.1901572116 31072937 PMC6561190

[B86] Kinuthia U. M. Wolf A. Langmann T. (2020). Microglia and inflammatory responses in diabetic retinopathy. Front. Immunol. 11, 564077. 10.3389/fimmu.2020.564077 33240260 PMC7681237

[B87] Kitaoka Y. Kitaoka Y. Kwong J. M. Ross-Cisneros F. N. Wang J. Tsai R. K. (2006). TNF-alpha-induced optic nerve degeneration and nuclear factor-kappaB p65. Invest Ophthalmol. Vis. Sci. 47 (4), 1448–1457. 10.1167/iovs.05-0299 16565378

[B88] Kohno H. Maeda T. Perusek L. Pearlman E. Maeda A. (2014). CCL3 production by microglial cells modulates disease severity in murine models of retinal degeneration. J. Immunol. 192 (8), 3816–3827. 10.4049/jimmunol.1301738 24639355 PMC4123815

[B89] Laffer B. Bauer D. Wasmuth S. Busch M. Jalilvand T. V. Thanos S. (2019). Loss of IL-10 promotes differentiation of microglia to a M1 phenotype. Front. Cell Neurosci. 13, 430. 10.3389/fncel.2019.00430 31649508 PMC6794388

[B90] Lee S. Chung W. G. Jeong H. Cui G. Kim E. Lim J. A. (2024). Electrophysiological analysis of retinal organoid development using 3D microelectrodes of liquid metals. Adv. Mater 36 (35), e2404428. 10.1002/adma.202404428 38896876

[B91] Lemaitre C. Thillaye-Goldenberg B. Naud M. C. de Kozak Y. (2001). The effects of intraocular injection of interleukin-13 on endotoxin-induced uveitis in rats. Invest Ophthalmol. Vis. Sci. 42 (9), 2022–2030. 11481267

[B92] Liao C. Xu J. Chen Y. Ip N. Y. (2021). Retinal dysfunction in Alzheimer's disease and implications for biomarkers. Biomolecules 11 (8), 1215. 10.3390/biom11081215 34439882 PMC8394950

[B93] Lin Y. T. Seo J. Gao F. Feldman H. M. Wen H. L. Penney J. (2018). APOE4 causes widespread molecular and cellular alterations associated with Alzheimer's disease phenotypes in human iPSC-derived brain cell types. Neuron 98 (6), 1141–1154. 10.1016/j.neuron.2018.05.008 29861287 PMC6023751

[B94] Lipski D. A. Dewispelaere R. Foucart V. Caspers L. E. Defrance M. Bruyns C. (2017). MHC class II expression and potential antigen-presenting cells in the retina during experimental autoimmune uveitis. J. Neuroinflammation 14 (1), 136. 10.1186/s12974-017-0915-5 28720143 PMC5516361

[B95] Liu Y. Sheng J. Y. Yang C. F. Ding J. Chan Y. S. (2023). A decade of liver organoids: advances in disease modeling. Clin. Mol. Hepatol. 29 (3), 643–669. 10.3350/cmh.2022.0428 36880210 PMC10366802

[B96] Low L. A. Mummery C. Berridge B. R. Austin C. P. Tagle D. A. (2021). Organs-on-chips: into the next decade. Nat. Rev. Drug Discov. 20 (5), 345–361. 10.1038/s41573-020-0079-3 32913334

[B97] Lowe A. Harris R. Bhansali P. Cvekl A. Liu W. (2016). Intercellular adhesion-dependent cell survival and ROCK-regulated actomyosin-driven forces mediate self-formation of a retinal organoid. Stem Cell Rep. 6 (5), 743–756. 10.1016/j.stemcr.2016.03.011 PMC4939656 27132890

[B98] Lukowski S. W. Lo C. Y. Sharov A. A. Nguyen Q. Fang L. Hung S. S. (2019). A single-cell transcriptome atlas of the adult human retina. Embo J. 38 (18), e100811. 10.15252/embj.2018100811 31436334 PMC6745503

[B99] Maisonneuve B. G. C. Batut A. Varela C. Vieira J. Gleyzes M. Rontard J. (2021). Neurite growth kinetics regulation through hydrostatic pressure in a novel triangle-shaped neurofluidic system. bioRxiv. 10.1101/2021.03.23.436675

[B100] Marcinkowska A. Wolska N. Luzak B. Cisiecki S. Marcinkowski K. Rozalski M. (2022). Platelet-derived procoagulant microvesicles are elevated in patients with retinal vein occlusion (RVO). J. Clin. Med. 11 (17), 5099. 10.3390/jcm11175099 36079028 PMC9457368

[B101] Martinelli I. Tayebati S. K. Tomassoni D. Nittari G. Roy P. Amenta F. (2022). Brain and retinal organoids for disease modeling: the importance of *in vitro* blood-brain and retinal barriers studies. Cells 11 (7), 1120. 10.3390/cells11071120 35406683 PMC8997725

[B102] Mathivanan I. Trepp C. Brunold C. Baerlocher G. Enzmann V. (2015). Retinal differentiation of human bone marrow-derived stem cells by co-culture with retinal pigment epithelium *in vitro* . Exp. Cell Res. 333 (1), 11–20. 10.1016/j.yexcr.2015.02.001 25724900

[B103] McMenamin P. G. Saban D. R. Dando S. J. (2019). Immune cells in the retina and choroid: two different tissue environments that require different defenses and surveillance. Prog. Retin Eye Res. 70, 85–98. 10.1016/j.preteyeres.2018.12.002 30552975 PMC7321801

[B104] Miller A. J. Dye B. R. Ferrer-Torres D. Hill D. R. Overeem A. W. Shea L. D. (2019). Generation of lung organoids from human pluripotent stem cells *in vitro* . Nat. Protoc. 14 (2), 518–540. 10.1038/s41596-018-0104-8 30664680 PMC6531049

[B105] Mills S. A. Jobling A. I. Dixon M. A. Bui B. V. Vessey K. A. Phipps J. A. (2021). Fractalkine-induced microglial vasoregulation occurs within the retina and is altered early in diabetic retinopathy. Proc. Natl. Acad. Sci. U. S. A. 118 (51), e2112561118. 10.1073/pnas.2112561118 34903661 PMC8713803

[B106] Mochizuki M. Sugita S. Kamoi K. (2013). Immunological homeostasis of the eye. Prog. Retin Eye Res. 33, 10–27. 10.1016/j.preteyeres.2012.10.002 23108335

[B107] Murenu E. Gerhardt M. J. Biel M. Michalakis S. (2022). More than meets the eye: the role of microglia in healthy and diseased retina. Front. Immunol. 13, 1006897. 10.3389/fimmu.2022.1006897 36524119 PMC9745050

[B108] Nakano T. Ando S. Takata N. Kawada M. Muguruma K. Sekiguchi K. (2012). Self-formation of optic cups and storable stratified neural retina from human ESCs. Cell Stem Cell 10 (6), 771–785. 10.1016/j.stem.2012.05.009 22704518

[B109] Narasipura S. D. Zayas J. P. Ash M. K. Reyes A. Shull T. Gambut S. (2024). HIV-1 infection promotes neuroinflammation and neuron pathogenesis in novel microglia-containing cerebral organoids. bioRxiv. 10.1101/2024.06.13.598579 PMC11808982 39930449

[B110] Naumovska E. Aalderink G. Wong Valencia C. Kosim K. Nicolas A. Brown S. (2020). Direct on-chip differentiation of intestinal tubules from induced pluripotent stem cells. Int. J. Mol. Sci. 21 (14), 4964. 10.3390/ijms21144964 32674311 PMC7404294

[B111] Nazarenko L. Didenko O. (2023). Road lighting and mesopic vision. Ukrainian Metrological Journal, 39–45. 10.24027/2306-7039.1.2023.282600

[B112] Nguyen H. Lee S. J. Li Y. (2022). Selective activation of the wnt-signaling pathway as a novel therapy for the treatment of diabetic retinopathy and other retinal vascular diseases. Pharmaceutics 14 (11), 2476. 10.3390/pharmaceutics14112476 36432666 PMC9697247

[B113] Niederkorn J. Y. (2019). The eye sees eye to eye with the immune system: the 2019 proctor lecture. Invest Ophthalmol. Vis. Sci. 60 (13), 4489–4495. 10.1167/iovs.19-28632 31661549 PMC6819053

[B114] Nieto-Aristizábal I. Mera J. J. Giraldo J. D. Lopez-Arevalo H. Tobón G. J. (2022). From ocular immune privilege to primary autoimmune diseases of the eye. Autoimmun. Rev. 21 (8), 103122. 10.1016/j.autrev.2022.103122 35667621

[B115] Noel G. Baetz N. W. Staab J. F. Donowitz M. Kovbasnjuk O. Pasetti M. F. (2017). A primary human macrophage-enteroid co-culture model to investigate mucosal gut physiology and host-pathogen interactions. Sci. Rep. 7, 45270. 10.1038/srep45270 28345602 PMC5366908

[B116] Ohteki T. Kawamura S. Onai N. (2021). Commitment to dendritic cells and monocytes. Int. Immunol. 33 (12), 815–819. 10.1093/intimm/dxab031 34134136

[B117] O'Keefe G. M. Nguyen V. T. Benveniste E. N. (1999). Class II transactivator and class II MHC gene expression in microglia: modulation by the cytokines TGF-beta, IL-4, IL-13 and IL-10. Eur. J. Immunol. 29 (4), 1275–1285. 10.1002/(SICI)1521-4141(199904)29:04<1275::AID-IMMU1275>3.0.CO;2-T 10229095

[B118] O'Koren E. G. Yu C. Klingeborn M. Wong A. Y. W. Prigge C. L. Mathew R. (2019). Microglial function is distinct in different anatomical locations during retinal homeostasis and degeneration. Immunity 50 (3), 723–737.e7. 10.1016/j.immuni.2019.02.007 30850344 PMC6592635

[B119] Okunuki Y. Mukai R. Nakao T. Tabor S. J. Butovsky O. Dana R. (2019). Retinal microglia initiate neuroinflammation in ocular autoimmunity. Proc. Natl. Acad. Sci. U. S. A. 116 (20), 9989–9998. 10.1073/pnas.1820387116 31023885 PMC6525481

[B120] O'Leary F. Campbell M. (2023). The blood-retina barrier in health and disease. Febs J. 290 (4), 878–891. 10.1111/febs.16330 34923749

[B121] Ormel P. R. Vieira de Sá R. van Bodegraven E. J. Karst H. Harschnitz O. Sneeboer M. A. M. (2018). Microglia innately develop within cerebral organoids. Nat. Commun. 9 (1), 4167. 10.1038/s41467-018-06684-2 30301888 PMC6177485

[B122] Pan D. Xia X. X. Zhou H. Jin S. Q. Lu Y. Y. Liu H. (2020). COCO enhances the efficiency of photoreceptor precursor differentiation in early human embryonic stem cell-derived retinal organoids. Stem Cell Res. Ther. 11 (1), 366. 10.1186/s13287-020-01883-5 32831148 PMC7444242

[B123] Papp D. Korcsmaros T. Hautefort I. (2024). Revolutionizing immune research with organoid-based co-culture and chip systems. Clin. Exp. Immunol. 218 (1), 40–54. 10.1093/cei/uxae004 38280212 PMC11404127

[B124] Park D. S. Kozaki T. Tiwari S. K. Moreira M. Khalilnezhad A. Torta F. (2023). iPS-cell-derived microglia promote brain organoid maturation via cholesterol transfer. Nature 623 (7986), 397–405. 10.1038/s41586-023-06713-1 37914940

[B125] Park J. Wetzel I. Marriott I. Dréau D. D'Avanzo C. Kim D. Y. (2018). A 3D human triculture system modeling neurodegeneration and neuroinflammation in Alzheimer's disease. Nat. Neurosci. 21 (7), 941–951. 10.1038/s41593-018-0175-4 29950669 PMC6800152

[B126] Paşca A. M. Park J. Y. Shin H. W. Qi Q. Revah O. Krasnoff R. (2019). Human 3D cellular model of hypoxic brain injury of prematurity. Nat. Med. 25 (5), 784–791. 10.1038/s41591-019-0436-0 31061540 PMC7020938

[B127] Pascual-Pasto G. McIntyre B. Giudice A. M. Alikarami F. Morrissey A. Matlaga S. (2024). Targeting GPC2 on intraocular and CNS metastatic retinoblastomas with local and systemic delivery of CAR T cells. Clin. Cancer Res. 30 (16), 3578–3591. 10.1158/1078-0432.CCR-24-0221 38864848 PMC11326963

[B128] Patel S. H. Lamba D. A. (2023). Factors affecting stem cell–based regenerative approaches in retinal degeneration. Annu. Rev. Vis. Sci. 9 (1), 155–175. 10.1146/annurev-vision-120222-012817 37713278

[B129] Penfold P. L. Madigan M. C. Provis J. M. (1991). Antibodies to human leucocyte antigens indicate subpopulations of microglia in human retina. Vis. Neurosci. 7 (4), 383–388. 10.1017/s0952523800004879 1751423

[B130] Phipps J. A. Vessey K. A. Brandli A. Nag N. Tran M. X. Jobling A. I. (2018). The role of angiotensin II/AT1 receptor signaling in regulating retinal microglial activation. Invest Ophthalmol. Vis. Sci. 59 (1), 487–498. 10.1167/iovs.17-22416 29368003

[B131] Qiao H. Lucas K. Stein-Streilein J. (2009). Retinal laser burn disrupts immune privilege in the eye. Am. J. Pathol. 174 (2), 414–422. 10.2353/ajpath.2009.080766 19147817 PMC2630551

[B132] Quadrato G. Nguyen T. Macosko E. Z. Sherwood J. L. Min Yang S. Berger D. R. (2017). Cell diversity and network dynamics in photosensitive human brain organoids. Nature 545 (7652), 48–53. 10.1038/nature22047 28445462 PMC5659341

[B133] Rashid K. Akhtar-Schaefer I. Langmann T. (2019). Microglia in retinal degeneration. Front. Immunol. 10, 1975. 10.3389/fimmu.2019.01975 31481963 PMC6710350

[B134] Rathnasamy G. Foulds W. S. Ling E. A. Kaur C. (2019). Retinal microglia - a key player in healthy and diseased retina. Prog. Neurobiol. 173, 18–40. 10.1016/j.pneurobio.2018.05.006 29864456

[B135] Read E. Jowett G. M. Coman D. Neves J. F. (2022). Co-culture of murine small intestine epithelial organoids with innate lymphoid cells. J. Vis. Exp. 181. 10.3791/63554 35404347

[B136] Richards D. J. Li Y. Kerr C. M. Yao J. Beeson G. C. Coyle R. C. (2020). Human cardiac organoids for the modelling of myocardial infarction and drug cardiotoxicity. Nat. Biomed. Eng. 4 (4), 446–462. 10.1038/s41551-020-0539-4 32284552 PMC7422941

[B137] Roberge F. G. de Smet M. D. Benichou J. Kriete M. F. Raber J. Hakimi J. (1998). Treatment of uveitis with recombinant human interleukin-13. Br. J. Ophthalmol. 82 (10), 1195–1198. 10.1136/bjo.82.10.1195 9924310 PMC1722394

[B138] Rutar M. Natoli R. Chia R. X. Valter K. Provis J. M. (2015). Chemokine-mediated inflammation in the degenerating retina is coordinated by Müller cells, activated microglia, and retinal pigment epithelium. J. Neuroinflammation 12, 8. 10.1186/s12974-014-0224-1 25595590 PMC4308937

[B139] Ryan S. K. Zelic M. Han Y. Teeple E. Chen L. Sadeghi M. (2023). Microglia ferroptosis is regulated by SEC24B and contributes to neurodegeneration. Nat. Neurosci. 26 (1), 12–26. 10.1038/s41593-022-01221-3 36536241 PMC9829540

[B140] Sabate-Soler S. Nickels S. L. Saraiva C. Berger E. Dubonyte U. Barmpa K. (2022). Microglia integration into human midbrain organoids leads to increased neuronal maturation and functionality. Glia 70 (7), 1267–1288. 10.1002/glia.24167 35262217 PMC9314680

[B141] Sahara M. (2023). Recent advances in generation of *in vitro* cardiac organoids. Int. J. Mol. Sci. 24 (7), 6244. 10.3390/ijms24076244 37047216 PMC10094119

[B142] Salter M. W. Beggs S. (2014). Sublime microglia: expanding roles for the guardians of the CNS. Cell 158 (1), 15–24. 10.1016/j.cell.2014.06.008 24995975

[B143] Sanie-Jahromi F. Mahmoudi A. Khalili M. R. Nowroozzadeh M. H. (2022). A review on the application of stem cell secretome in the protection and regeneration of retinal ganglion cells; a clinical prospect in the treatment of optic neuropathies. Curr. Eye Res. 47 (11), 1463–1471. 10.1080/02713683.2022.2103153 35876610

[B144] Santos A. M. Calvente R. Tassi M. Carrasco M. C. Martín-Oliva D. Marín-Teva J. L. (2008). Embryonic and postnatal development of microglial cells in the mouse retina. J. Comp. Neurol. 506 (2), 224–239. 10.1002/cne.21538 18022954

[B145] Schlotterer A. Kolibabka M. Lin J. Acunman K. Dietrich N. Sticht C. (2019). Methylglyoxal induces retinopathy-type lesions in the absence of hyperglycemia: studies in a rat model. Faseb J. 33 (3), 4141–4153. 10.1096/fj.201801146RR 30485119

[B146] Schmied V. Korkut-Demirbaş M. Venturino A. Siegert S. (2024). Microglia determine an immune-challenged environment and facilitate ibuprofen action in human retinal organoids. bioRxiv. 10.1101/2024.09.20.614136 PMC11966913 40181459

[B147] Schulz C. Gomez Perdiguero E. Chorro L. Szabo-Rogers H. Cagnard N. Kierdorf K. (2012). A lineage of myeloid cells independent of Myb and hematopoietic stem cells. Science 336 (6077), 86–90. 10.1126/science.1219179 22442384

[B148] Sennlaub F. Auvynet C. Calippe B. Lavalette S. Poupel L. Hu S. J. (2013). CCR2(+) monocytes infiltrate atrophic lesions in age-related macular disease and mediate photoreceptor degeneration in experimental subretinal inflammation in Cx3cr1 deficient mice. EMBO Mol. Med. 5 (11), 1775–1793. 10.1002/emmm.201302692 24142887 PMC3840491

[B149] Serra D. Mayr U. Boni A. Lukonin I. Rempfler M. Challet Meylan L. (2019). Self-organization and symmetry breaking in intestinal organoid development. Nature 569 (7754), 66–72. 10.1038/s41586-019-1146-y 31019299 PMC6544541

[B150] Shin J. H. Jeong J. Maher S. E. Lee H. W. Lim J. Bothwell A. L. M. (2021). Colon cancer cells acquire immune regulatory molecules from tumor-infiltrating lymphocytes by trogocytosis. Proc. Natl. Acad. Sci. U. S. A. 118 (48), e2110241118. 10.1073/pnas.2110241118 34819374 PMC8640789

[B151] Shinozaki Y. Kashiwagi K. Koizumi S. (2023). Astrocyte immune functions and glaucoma. Int. J. Mol. Sci. 24 (3), 2747. 10.3390/ijms24032747 36769067 PMC9916878

[B152] Shiraki N. Maruyama K. Hayashi R. Oguchi A. Murakawa Y. Katayama T. (2022). PAX6-positive microglia evolve locally in hiPSC-derived ocular organoids. Stem Cell Rep. 17 (2), 221–230. 10.1016/j.stemcr.2021.12.009 PMC8828554 35030319

[B153] Sierra A. Navascués J. Cuadros M. A. Calvente R. Martín-Oliva D. Ferrer-Martín R. M. (2014). Expression of inducible nitric oxide synthase (iNOS) in microglia of the developing quail retina. PLoS One 9 (8), e106048. 10.1371/journal.pone.0106048 25170849 PMC4149512

[B154] Silverman S. M. Kim B. J. Howell G. R. Miller J. John S. W. Wordinger R. J. (2016). C1q propagates microglial activation and neurodegeneration in the visual axis following retinal ischemia/reperfusion injury. Mol. Neurodegener. 11, 24. 10.1186/s13024-016-0089-0 27008854 PMC4806521

[B155] Silverman S. M. Wong W. T. (2018). Microglia in the retina: roles in development, maturity, and disease. Annu. Rev. Vis. Sci. 4, 45–77. 10.1146/annurev-vision-091517-034425 29852094

[B156] Simmons A. B. Merrill M. M. Reed J. C. Deans M. R. Edwards M. M. Fuerst P. G. (2016). Defective angiogenesis and intraretinal bleeding in mouse models with disrupted inner retinal lamination. Invest Ophthalmol. Vis. Sci. 57 (4), 1563–1577. 10.1167/iovs.15-18395 27046121 PMC4824390

[B157] Smirnova L. Hartung T. (2024). The promise and potential of brain organoids. Adv. Healthc. Mater 13 (21), e2302745. 10.1002/adhm.202302745 38252094

[B158] Smith A. M. Graham E. S. Feng S. X. Oldfield R. L. Bergin P. M. Mee E. W. (2013). Adult human glia, pericytes and meningeal fibroblasts respond similarly to IFNy but not to TGFβ1 or M-CSF. PLoS One 8 (12), e80463. 10.1371/journal.pone.0080463 24339874 PMC3855168

[B159] Spijkers X. M. Pasteuning-Vuhman S. Dorleijn J. C. Vulto P. Wevers N. R. Pasterkamp R. J. (2021). A directional 3D neurite outgrowth model for studying motor axon biology and disease. Sci. Rep. 11 (1), 2080. 10.1038/s41598-021-81335-z 33483540 PMC7822896

[B160] Sridhar A. Hoshino A. Finkbeiner C. R. Chitsazan A. Dai L. Haugan A. K. (2020). Single-cell transcriptomic comparison of human fetal retina, hPSC-derived retinal organoids, and long-term retinal cultures. Cell Rep. 30 (5), 1644–1659. 10.1016/j.celrep.2020.01.007 32023475 PMC7901645

[B161] Sterling J. K. Rajesh A. Droho S. Gong J. Wang A. L. Voigt A. P. (2024). Retinal perivascular macrophages regulate immune cell infiltration during neuroinflammation in mouse models of ocular disease. J. Clin. Invest 134 (20), e180904. 10.1172/JCI180904 39207852 PMC11473146

[B162] Su T. Liang L. Zhang L. Wang J. Chen L. Su C. (2022). Retinal organoids and microfluidic chip-based approaches to explore the retinitis pigmentosa with USH2A mutations. Front. Bioeng. Biotechnol. 10, 939774. 10.3389/fbioe.2022.939774 36185441 PMC9524156

[B163] Subramani M. Van Hook M. J. Qiu F. Ahmad I. (2023). Human retinal ganglion cells respond to evolutionarily conserved chemotropic cues for intra retinal guidance and regeneration. Stem Cells 41 (11), 1022–1036. 10.1093/stmcls/sxad061 37591511 PMC13032126

[B164] Šuligoj T. Vigsnæs L. K. Abbeele P. V. D. Apostolou A. Karalis K. Savva G. M. (2020). Effects of human milk oligosaccharides on the adult gut microbiota and barrier function. Nutrients 12 (9), 2808. 10.3390/nu12092808 32933181 PMC7551690

[B165] Sun X. Cui Z. Liang Y. Duan C. Chan H. F. Mao S. (2023). One-stop assembly of adherent 3D retinal organoids from hiPSCs based on 3D-printed derived PDMS microwell platform. Biofabrication 15 (3), 035005. 10.1088/1758-5090/acc761 36963105

[B166] Sun X. Y. Ju X. C. Li Y. Zeng P. M. Wu J. Zhou Y. Y. (2022). Generation of vascularized brain organoids to study neurovascular interactions. Elife 11, e76707. 10.7554/eLife.76707 35506651 PMC9246368

[B167] Takebe T. Zhang B. Radisic M. (2017). Synergistic engineering: organoids meet organs-on-a-chip. Cell Stem Cell 21 (3), 297–300. 10.1016/j.stem.2017.08.016 28886364

[B168] Tang Y. Cheng Y. Wang S. Wang Y. Liu P. Wu H. (2022). Review: the development of risk factors and cytokines in retinal vein occlusion. Front. Med. (Lausanne) 9, 910600. 10.3389/fmed.2022.910600 35783660 PMC9240302

[B169] Taylor J. Sellin J. Kuerschner L. Krähl L. Majlesain Y. Förster I. (2020). Generation of immune cell containing adipose organoids for *in vitro* analysis of immune metabolism. Sci. Rep. 10 (1), 21104. 10.1038/s41598-020-78015-9 33273595 PMC7713299

[B170] Treveil A. Sudhakar P. Matthews Z. J. Wrzesiński T. Jones E. J. Brooks J. (2020). Regulatory network analysis of Paneth cell and goblet cell enriched gut organoids using transcriptomics approaches. Mol. Omics 16 (1), 39–58. 10.1039/c9mo00130a 31819932

[B171] Usui-Ouchi A. Giles S. Harkins-Perry S. Mills E. A. Bonelli R. Wei G. (2023). Integrating human iPSC-derived macrophage progenitors into retinal organoids to generate a mature retinal microglial niche. Glia 71 (10), 2372–2382. 10.1002/glia.24428 37335016

[B172] Usui-Ouchi A. Usui Y. Kurihara T. Aguilar E. Dorrell M. I. Ideguchi Y. (2020). Retinal microglia are critical for subretinal neovascular formation. JCI Insight 5 (12), e137317. 10.1172/jci.insight.137317 32437334 PMC7406258

[B173] Verbakel S. K. van Huet R. A. C. Boon C. J. F. den Hollander A. I. Collin R. W. J. Klaver C. C. W. (2018). Non-syndromic retinitis pigmentosa. Prog. Retin Eye Res. 66, 157–186. 10.1016/j.preteyeres.2018.03.005 29597005

[B174] Wang J. Ohno-Matsui K. Yoshida T. Shimada N. Ichinose S. Sato T. (2009). Amyloid-beta up-regulates complement factor B in retinal pigment epithelial cells through cytokines released from recruited macrophages/microglia: another mechanism of complement activation in age-related macular degeneration. J. Cell Physiol. 220 (1), 119–128. 10.1002/jcp.21742 19277984

[B175] Wang M. Ma W. Zhao L. Fariss R. N. Wong W. T. (2011). Adaptive Müller cell responses to microglial activation mediate neuroprotection and coordinate inflammation in the retina. J. Neuroinflammation 8, 173. 10.1186/1742-2094-8-173 22152278 PMC3251543

[B176] Wang S. K. Cepko C. L. (2022). Targeting microglia to treat degenerative eye diseases. Front. Immunol. 13, 843558. 10.3389/fimmu.2022.843558 35251042 PMC8891158

[B177] Wang S. K. Xue Y. Cepko C. L. (2021). Augmentation of CD47/SIRPα signaling protects cones in genetic models of retinal degeneration. JCI Insight 6 (16), e150796. 10.1172/jci.insight.150796 34197341 PMC8409989

[B178] Wang X. Wang T. Lam E. Alvarez D. Sun Y. (2023). Ocular vascular diseases: from retinal immune privilege to inflammation. Int. J. Mol. Sci. 24 (15), 12090. 10.3390/ijms241512090 37569464 PMC10418793

[B179] Watson A. Lako M. (2023). Retinal organoids provide unique insights into molecular signatures of inherited retinal disease throughout retinogenesis. J. Anat. 243 (2), 186–203. 10.1111/joa.13768 36177499 PMC10335378

[B180] Wevers N. R. Kasi D. G. Gray T. Wilschut K. J. Smith B. van Vught R. (2018). A perfused human blood-brain barrier on-a-chip for high-throughput assessment of barrier function and antibody transport. Fluids Barriers CNS 15 (1), 23. 10.1186/s12987-018-0108-3 30165870 PMC6117964

[B181] Wu Q. Liu J. Wang X. Feng L. Wu J. Zhu X. (2020). Organ-on-a-chip: recent breakthroughs and future prospects. Biomed. Eng. Online 19 (1), 9. 10.1186/s12938-020-0752-0 32050989 PMC7017614

[B182] Wu Y. Hirschi K. K. (2020). Tissue-resident macrophage development and function. Front. Cell Dev. Biol. 8, 617879. 10.3389/fcell.2020.617879 33490082 PMC7820365

[B183] Wu Y. Zhou Y. Qin X. Liu Y. (2021). From cell spheroids to vascularized cancer organoids: microfluidic tumor-on-a-chip models for preclinical drug evaluations. Biomicrofluidics 15 (6), 061503. 10.1063/5.0062697 34804315 PMC8589468

[B184] Xu H. Dawson R. Forrester J. V. Liversidge J. (2007). Identification of novel dendritic cell populations in normal mouse retina. Invest Ophthalmol. Vis. Sci. 48 (4), 1701–1710. 10.1167/iovs.06-0697 17389502 PMC2446435

[B185] Xu H. Forrester J. V. Liversidge J. Crane I. J. (2003). Leukocyte trafficking in experimental autoimmune uveitis: breakdown of blood-retinal barrier and upregulation of cellular adhesion molecules. Invest Ophthalmol. Vis. Sci. 44 (1), 226–234. 10.1167/iovs.01-1202 12506079

[B186] Xu J. Yu S.-J. Jin Z.-B. (2024b). Assembling retinal organoids with microglia. J. Vis. Exp. (209). 10.3791/67016 39141532

[B187] Xu J. Yu S. J. Sun S. Li Y. P. Zhang X. Jin K. (2024a). Enhanced innate responses in microglia derived from retinoblastoma patient-specific iPSCs. Glia 72 (5), 872–884. 10.1002/glia.24507 38258347

[B188] Xu K. Nie W. Tong Q. Ma L. Liu J. Wang Y. (2022). Analysis of progress characteristics of retinoblastoma based on single cell transcriptome sequencing. Sheng Wu Gong Cheng Xue Bao 38 (10), 3809–3824. 10.13345/j.cjb.220491 36305411

[B189] Yin S. Cui Y. Jiao W. Zhao B. (2022). Potential prognostic indicators for patients with retinal vein occlusion. Front. Med. (Lausanne) 9, 839082. 10.3389/fmed.2022.839082 35692537 PMC9174432

[B190] Yousef Yengej F. A. Jansen J. Rookmaaker M. B. Verhaar M. C. Clevers H. (2020). Kidney organoids and tubuloids. Cells 9 (6), 1326. 10.3390/cells9061326 32466429 PMC7349753

[B191] Zabel M. K. Zhao L. Zhang Y. Gonzalez S. R. Ma W. Wang X. (2016). Microglial phagocytosis and activation underlying photoreceptor degeneration is regulated by CX3CL1-CX3CR1 signaling in a mouse model of retinitis pigmentosa. Glia 64 (9), 1479–1491. 10.1002/glia.23016 27314452 PMC4958518

[B192] Zeng H. Y. Green W. R. Tso M. O. (2008). Microglial activation in human diabetic retinopathy. Arch. Ophthalmol. 126 (2), 227–232. 10.1001/archophthalmol.2007.65 18268214

[B193] Zhan L. (2023). Frontiers in understanding the pathological mechanism of diabetic retinopathy. Med. Sci. Monit. 29, e939658. 10.12659/MSM.939658 37307243 PMC10274216

[B194] Zhang X. Jin Z. B. (2021). Directed induction of retinal organoids from human pluripotent stem cells. J. Vis. Exp. 170. 10.3791/62298 33970142

[B195] Zhang Y. Wong W. T. (2021). Innate immunity in age-related macular degeneration. Adv. Exp. Med. Biol. 1256, 121–141. 10.1007/978-3-030-66014-7_5 33848000

[B196] Zhao F. Yu J. S. (2024). Overview of dendritic cells and related pathways in autoimmune uveitis. Open Life Sci. 19 (1), 20220887. 10.1515/biol-2022-0887 39290500 PMC11406227

[B197] Zhao L. Zabel M. K. Wang X. Ma W. Shah P. Fariss R. N. (2015). Microglial phagocytosis of living photoreceptors contributes to inherited retinal degeneration. EMBO Mol. Med. 7 (9), 1179–1197. 10.15252/emmm.201505298 26139610 PMC4568951

[B198] Zhao X. Sun R. Luo X. Wang F. Sun X. (2021b). The interaction between microglia and macroglia in glaucoma. Front. Neurosci. 15, 610788. 10.3389/fnins.2021.610788 34121982 PMC8193936

[B199] Zhao X. Xu Z. Xiao L. Shi T. Xiao H. Wang Y. (2021a). Review on the vascularization of organoids and organoids-on-a-chip. Front. Bioeng. Biotechnol. 9, 637048. 10.3389/fbioe.2021.637048 33912545 PMC8072266

[B200] Zhao Y. Wu Y. Islam K. Paul R. Zhou Y. Qin X. (2024). Microphysiologically engineered vessel-tumor model to investigate vascular transport dynamics of immune cells. ACS Appl. Mater. and Interfaces 16, 22839–22849. 10.1021/acsami.4c00391 38652824 PMC11082852

[B201] Zheng K. Huang H. Yang J. Qiu M. (2022). Origin, molecular specification, and stemness of astrocytes. Dev. Neurobiol. 82 (2), 149–159. 10.1002/dneu.22863 35006642

[B202] Zhong X. Gutierrez C. Xue T. Hampton C. Vergara M. N. Cao L. H. (2014). Generation of three-dimensional retinal tissue with functional photoreceptors from human iPSCs. Nat. Commun. 5, 4047. 10.1038/ncomms5047 24915161 PMC4370190

[B203] Zhu X. Hong J. Zhou X. (2023). Biological immune mechanism of retina. Front. Biosci. Landmark Ed. 28 (12), 363. 10.31083/j.fbl2812363 38179761

[B204] Zinkernagel M. S. Chinnery H. R. Ong M. L. Petitjean C. Voigt V. McLenachan S. (2013). Interferon γ-dependent migration of microglial cells in the retina after systemic cytomegalovirus infection. Am. J. Pathol. 182 (3), 875–885. 10.1016/j.ajpath.2012.11.031 23313136

[B205] Zou T. Gao L. Zeng Y. Li Q. Li Y. Chen S. (2019). Organoid-derived C-Kit(+)/SSEA4(-) human retinal progenitor cells promote a protective retinal microenvironment during transplantation in rodents. Nat. Commun. 10 (1), 1205. 10.1038/s41467-019-08961-0 30872578 PMC6418223

